# Confounding factors in assessing the enriched expression of somatic mutant alleles in bulk tumor samples

**DOI:** 10.1101/gr.281003.125

**Published:** 2026-04

**Authors:** Kohei Hagiwara, Andrew Thrasher, Nadezhda V. Terekhanova, Jinghui Zhang

**Affiliations:** Department of Computational Biology, St. Jude Children's Research Hospital, Memphis, Tennessee 38105, USA

## Abstract

Allele-specific expression (ASE) of somatic mutations can be caused by *cis*-activation of the mutant allele or silencing of the wild-type allele and has been investigated by examining the enrichment of mutant allele in RNA relative to DNA. Here we show that this mutation-based approach can be confounded by gene expression differences in tumor and normal cells that coexist in most bulk tumor samples. We model mutant allele expression by incorporating tumor/normal expression difference, mutant allele dosage, tumor purity, and nonsense-mediated decay (NMD) efficiency, projecting that such enrichments can occur without ASE. This confounding effect is exacerbated with low tumor purity and is dependent on mutant allele dosage for NMD-triggering mutations. The model predictions are validated by a pancancer bulk tumor analysis with somatic insertions/deletions (indels) from 9101 The Cancer Genome Atlas (TCGA) samples. A single-cell analysis in five cutaneous squamous cell carcinomas demonstrates the robustness of this model to intratumor heterogeneity. As a byproduct of this confounding effect, we evaluate whether the inverse relationship between mutant allele enrichment in RNA and tumor purity could be leveraged to complement DNA-based somatic mutation detection in low purity samples. Indeed, our de novo somatic indel calling from TCGA RNA-seq increases the TCGA driver indel repertoire by ∼14%, especially in samples with purity less than 0.4, including actionable *EGFR* indels in lung adenocarcinoma and *FLT3* in acute myeloid leukemia. Our study not only reveals confounders in somatic mutant ASE analysis but also demonstrates their utility in RNA-based mutation calling.

In a diploid genome, allele-specific expression (ASE) refers to a phenomenon of preferential expression of one allele over the other in a cell, which can unveil *cis*-acting regulatory mechanisms of attenuating (including silencing) or enhancing (including activation) the transcription of one specific allele (Ge et al. 2009). A conventional approach to characterize ASE is to test if the variant allele fraction (VAF) in RNA (VAF^RNA^) is significantly deviated from its expected VAF in DNA (VAF^DNA^). In noncancerous tissues, the analysis of the allelic imbalance of germline heterozygous single-nucleotide polymorphisms (SNPs) in RNA, which measures a significant deviation from the expected VAF^DNA^ of 0.5, has led to the identification of genes or regions that exhibit ASE (Castel et al. 2015). However, when applying the same technique to cancerous tissues (Mayba et al. 2014; PCAWG Transcriptome Core Group 2020; Robles-Espinoza et al. 2021), these imbalances were mostly (84.3%) attributed to somatically acquired copy-number alterations (CNAs) in the tumor DNA (PCAWG Transcriptome Core Group 2020).

More recent studies have attempted to characterize ASE events independent of somatic CNA by focusing on the ASE of somatic mutations (Liu et al. 2018; Palou-Márquez et al. 2024; Black et al. 2025; Bollas et al. 2025). This approach tests the biased expression of the mutant allele by directly comparing a mutation's VAF^RNA^ or comparable metrics to those in the tumor DNA genome, in which the influence of CNAs is accounted for, and a significant deviation is attributed to ASE. Such interpretation is valid under the condition that gene expression level is comparable in tumor cells and “contaminating” normal cells that coexist in bulk tumor samples. However, it is currently unclear how the difference in expression levels between tumor and normal cells affects the assessment of somatic mutant allele expression relative to its VAF^DNA^. Tumor and normal tissues are known to exhibit differential gene expressions (Zhang et al. 1997). For example, the tumor suppressor gene (TSG) *CDKN2A* was noted to have significantly higher expression in tumor tissues compared with the matching normal tissues acquired from cancer patients (Chen et al. 2021). Furthermore, tumor cells can undergo hypertranscription, a global change in transcription activity (Lin et al. 2012; Bowry et al. 2021; Zatzman et al. 2022), which can also result in expression difference.

Here, we present a theoretical framework to investigate the impact of expression difference on the somatic VAF imbalance in bulk RNA-seq samples. Our model shows that somatic mutant alleles can be enriched in RNA relative to DNA owing to expression differences in tumor and normal cells without ASE and that this relative enrichment becomes more pronounced with lower tumor purity. Although this effect can confound the ASE analysis of somatic mutations, we propose that it can be leveraged to sensitively detect mutations from RNA-seq in low-tumor-purity samples.

## Results

### Study design

**Figure 1.**
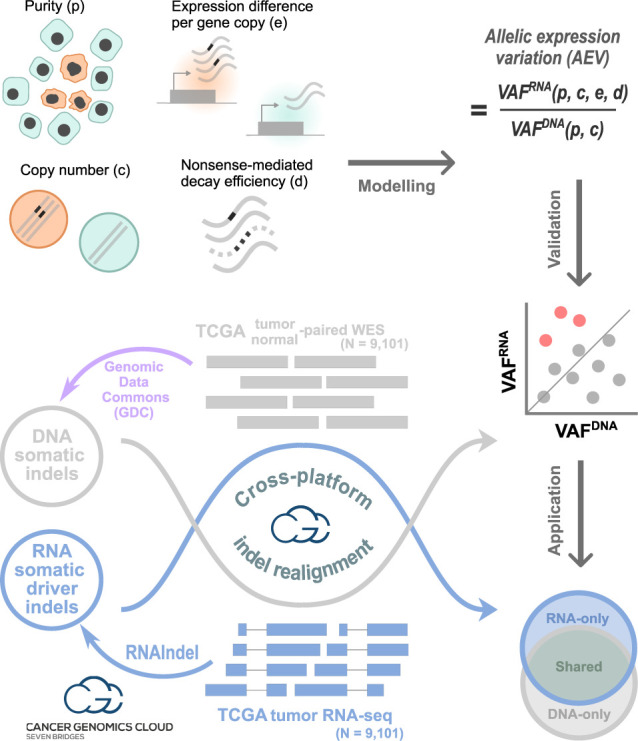
Study design with three main workflows. In *modeling*, the purity, copy number, expression level per copy in tumor relative to normal, and nonsense-mediated decay (NMD) efficiency were used to model the VAF ratio of RNA to DNA (i.e., VAF_RNA_/VAF_DNA_), which is termed allelic expression variation (AEV). Theoretical and simulation studies were conducted to identify conditions for AEV > 1 (elevated AEV). In *validation*, DNA somatic indels identified by the Genomic Data Commons's (GDC) multicaller platform in the TCGA data set (purple workflow) were used to validate the model for elevated AEV (red dots). Reads supporting indels were realigned in both WES and RNA-seq to accurately calculate AEV. In *application*, confounding effects leading to elevated AEV can be exploited to enhance mutation calling from RNA-seq. This was tested for somatic driver indel detection by calling from RNA-seq with WES read support to complement the TCGA indel set (Venn diagram). Indels in each Venn section were examined for AEV and purity along with potential clinical implications. Major computational analyses for validation and application were performed on the Cancer Genomics Cloud.

Tumor purity, somatic CNA, expression difference per gene copy in tumor relative to normal tissues, and nonsense-mediated decay (NMD) efficiency were used as parameters to model the ratio of VAF^RNA^ to VAF^DNA^, referred as allelic expression variation (AEV) (Fig. 1, *modeling*). Theoretical and simulation analyses of this model were performed to investigate whether unbalanced AEV (i.e., AEV ≠ 1) could be affected by these parameters. By focusing on mutations with elevated allelic expression (i.e., AEV > 1), we validated this theoretical model using 9101 The Cancer Genome Atlas (TCGA) (Cancer Genome Atlas Research Network 2013) samples with whole-exome sequencing (WES) and RNA-seq data sets available on the Cancer Genomics Cloud (CGC) (Lau et al. 2017) (Fig. 1, *validation* workflow). In this analysis, we used publicly available somatic insertions/deletions (indels) identified in WES by the Genomic Data Commons (GDC) (Zhang et al. 2021) multicaller pipeline. The use of indels enables the ability to assess the effect of NMD in an unbiased manner, whereas premature termination codons (PTCs) by nonsense single-nucleotide variants (SNVs) can be constrained to certain genomic contexts (e.g., TCA > TGA and hotspots). Although the model parameters can confound the detection of true ASE, elevated AEV can be exploited for RNA-based somatic mutation detection to complement DNA-based approach. Using the TCGA RNA-seq data, we performed de novo indel detection because DNA-based indel detection is known to have a limited sensitivity of 40%–50% (Fig. 1, *application* workflow; Alioto et al. 2015; The ICGC/TCGA Pan-Cancer Analysis of Whole Genomes Consortium 2020). We also evaluated the effectiveness of this approach for mutations rated actionable by the COSMIC Actionability database (Sondka et al. 2024) to explore the potential for clinical impact.

### Modeling AEV for somatic mutation

**Figure 2.**
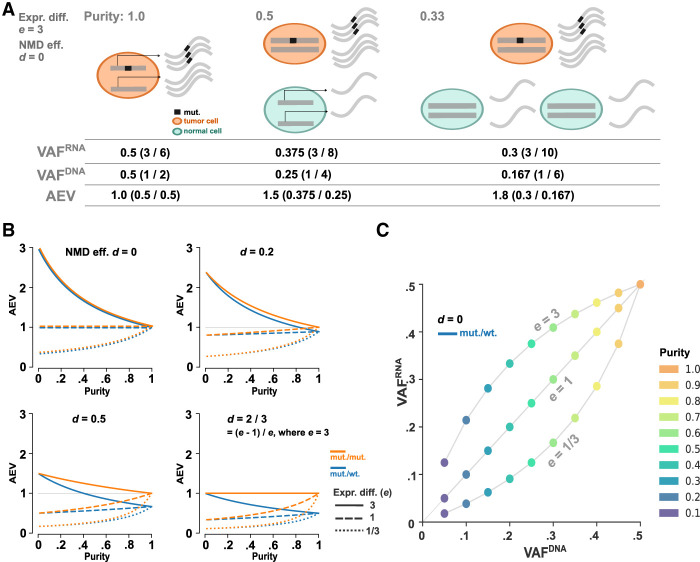
Simulation on AEV in absence of ASE. (*A*) Illustration of elevated AEV under the scenario that gene expression in the tumor is threefold of that in normal cells (*e* = 3) with the absence of NMD (*d* = 0). In tumor cells, three copies of the mutant and wild-type alleles are transcribed, whereas a single copy of the alleles is transcribed in normal cells. The VAF^RNA^, VAF^DNA^, and the ratio of VAF^RNA^ to VAF^DNA^ (AEV) projected from tumor purity at 1.0, 0.5, and 0.33 are presented *below*. (*B*) Simulation of the AEV model for heterozygous (blue) and homozygous (orange) mutation in a diploid region with varying degrees of tumor purity and NMD efficiencies. (*C*) Simulated VAF^DNA^ versus VAF^RNA^ under three expression difference scenarios for purity ranging from 0.1 to 1.0. The simulation is performed for NMD-insensitive, heterozygous mutation.

We considered a somatic mutation with a total copy number of *c* represented by *m* and *w* copies of mutant and wild-type alleles per cell, respectively (i.e., copy number *c* = *m* + *w*) and modeled its DNA VAF in a bulk sample (VAF^DNA^; Equation 1 in the Methods). We also modeled the mutation's VAF in RNA, in which the gene expression per copy is *e* times different between tumor and normal, and the mutant allele is degraded at NMD efficiency *d* (VAF^RNA^; Equation 2). The ratio of VAF^RNA^ to VAF^DNA^, defined as AEV (Equation 3) (for illustrative examples, see Fig. 2A; Supplemental Fig. S1), is used to show enriched (elevated AEV) or depleted (reduced AEV) mutant allele representation in RNA-seq. Our model projects that higher expression in the tumor per gene copy (*e* > 1) is required for elevated AEV (Equation 4), and we simulated the model over tumor purity in copy number *c* = 2 (diploid) at various NMD efficiencies (Fig. 2B). For mutations that do not trigger NMD (i.e., *d* = 0), no expression variation is expected (i.e., AEV = 1) when the gene expression levels per copy are identical in tumor and normal cells (i.e., *e* = 1) in contrast to the elevated and reduced AEV expected for *e* > 1 and *e* < 1, respectively. Further, this effect on AEV is more prominent with lower purity owing to a different rate of VAF^RNA^ reduction compared with that of VAF^DNA^ (Fig. 2C). In absence of NMD, the mutant allele dosage has no effect on AEV (Equation 5) (Fig. 2B, *d* = 0). However, with *d* > 0, enriched mutant allele dosage owing to amplification of the mutant allele or deletion of the wild-type allele (loss of heterozygosity [LOH]) results in higher AEV (e.g., mut./mut. vs. mut./wt. in the three panels with *d* > 0 in Fig. 2B). Although higher NMD efficiency decreases AEV, elevated AEV is still detectable if the efficiency is smaller than (*e* − 1)/*e* (Equation 6) (see Fig. 2B, *d* = 2/3). Similar results are obtained in other copy numbers (Supplemental Fig. S2) and also for subclonal mutation/CNA events (Equation 8 in the Methods) (Supplemental Fig. S3).

In summary, under the condition in which the gene expression per copy is higher in tumor than in normal cells (i.e., *e* > 1), elevated AEV (i.e., AEV > 1) can be detected in absence of ASE and the pattern is more pronounced with reduced tumor purity. Although NMD decreases AEV, it can be retained at a higher level with enriched mutant allele dosage.

### Elevated AEV in somatic truncation indels in TCGA

To empirically validate the AEV model, we used 106,157 somatic coding indels on the autosomal chromosomes identified by NCI GDC. These indels were annotated for amino acid changes, and the underlying genes were categorized as TSGs, oncogenes, or nondriver genes (Methods). Truncation indels (frameshifts and in-frame insertions of stop codons) accounted for the vast majority of all somatic indels (92.93% or 98,649/106,157), and those in driver genes primarily disrupted TSGs (93.05%, 4724 truncations in TSGs of 5077 in driver genes). In TSGs, truncations are more frequently accompanied by LOH, conforming to the two-hit hypothesis of tumorigenesis (Knudson 1971). Therefore, this indel class is well suited for testing the predicted effects of LOH and NMD efficiency.

**Figure 3.**
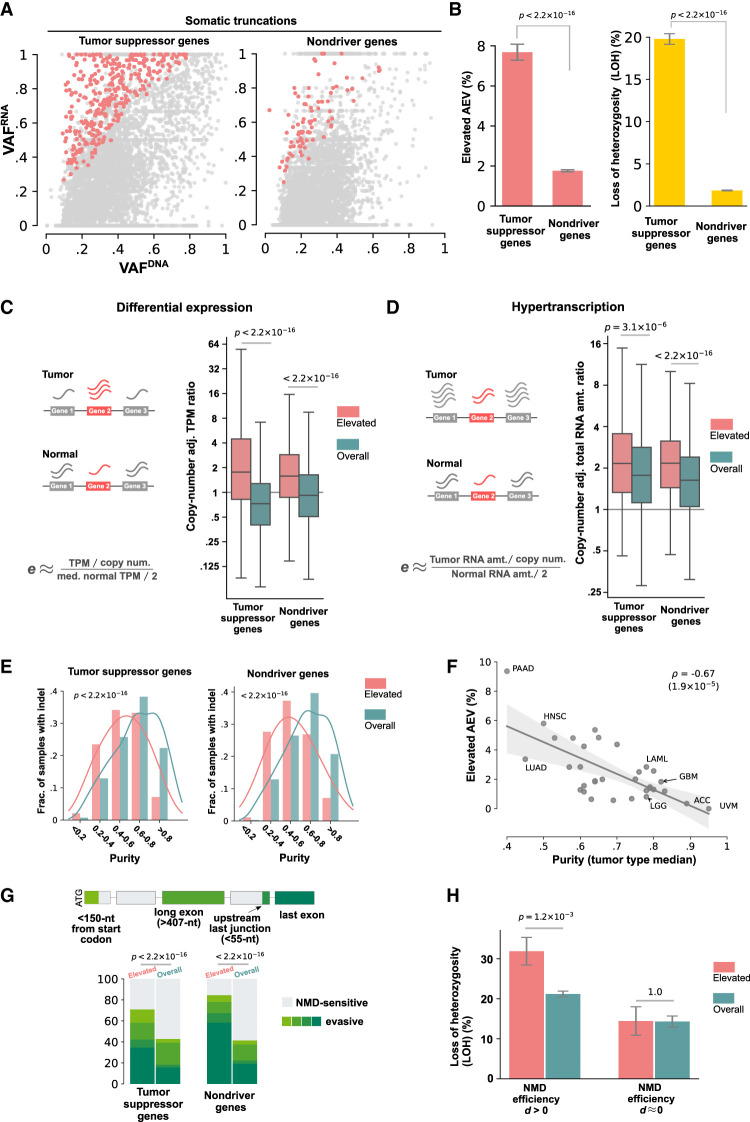
Evaluation of the AEV model using somatic truncation indels in TCGA samples. (*A*) Comparison of elevated AEV indels (AEV > 1.2 with FDR < 0.01) in tumor-suppressor genes (TSGs) and nondriver genes. Scatter plots showing VAF^DNA^ (*x*-axis) and VAF^RNA^ (*y*-axis) for indels in TSGs and nondrivers with elevated AEVs highlighted in red dots. Nondriver indels were down-sampled to match the TSG indel size for visualization. (*B*) The prevalence of elevated AEV in TSGs and nondrivers was compared by Fisher's exact test using the full data set, which contained 4450 TSG and 69,840 nondriver indels, respectively (*left*). The involvement of LOH at indel loci was also compared by Fisher's exact test (*right*). (*C*,*D*) Evaluation of expression difference in tumor versus normal on elevated AEV is shown for differential expression (*C*) and for global upregulation in transcription activity (hypertranscription; *D*). For measuring differential expressions in *C*, relative expression per copy (*e*) was surrogated by dividing each tumor sample's TPM with the median TPM in tissue-matched normal samples adjusted for copy number. Box plots compare the distribution of the TMP ratio for samples harboring elevated AEV indels (*elevated*) versus all indels in the corresponding gene category (*overall*) with *P-*values by Mann–Whitney *U* test. The expression ratio plotted in *D* was based on the RNA amount ratio estimated by Zatzman et al. (2022). (*E*,*F*) The effect of tumor purity on elevated AEV. (*E*) Comparison of tumor-purity distribution in samples harboring elevated indels (*elevated*) versus all indel-containing samples (*overall*) by Mann–Whitney *U* test. (*F*) Correlation of elevated AEV prevalence (*y*-axis) in a tumor type with the median purity of the tumor type (*x*-axis) by linear regression. (PAAD) Pancreatic adenocarcinoma; (HNSC) head and neck squamous cell carcinoma; (LUAD) lung adenocarcinoma; (LAML) acute myeloid leukemia; (GBM) glioblastoma multiforme; (LGG) lower-grade glioma; (ACC) adrenocortical carcinoma; (UVM) uveal melanoma. (*G*,*H*) The effect of NMD on elevated AEV is shown in *G* for the comparison of NMD features by χ^2^ test (*G*) and in for comparison of the prevalence of LOH by Fisher's exact test stratified by NMD efficiency (*d* > 0 vs. *d* ≈ 0; *H*).

First, we quantified indel VAFs in both WES and RNA-seq using mutant and wild-type read counts extracted by indelPost (Hagiwara et al. 2022), as this tool outperformed those used by GDC in the benchmarking of indel VAF with serially diluted tumor DNA samples and expressed indels in RNA-seq (Methods) (Supplemental Fig. S4; Supplemental Tables S1, S2). When NMD occurs at a high efficiency, the transcript harboring the truncation allele may be degraded and thus undetectable, but the wild-type transcript can be present in the transcriptome. To capture this scenario, we required the indel locus to be covered with one or more RNA-seq reads regardless of mutation status, resulting in inclusion of 4450 truncations in TSGs and 69,840 in nondriver genes (Supplemental Table S3). Compared with that of nondriver genes, elevated AEV was four times as prevalent in TSGs (7.69% vs. 1.76%, Fisher's exact test *P* < 2.2 × 10^−16^) (Fig. 3A,B, left), in which LOH was 11 times more frequent (19.78% in TSGs vs. 1.84% in nondrivers, Fisher's exact test *P* < 2.2 × 10^−16^) (Fig. 3B, right).

We then examined whether the elevated AEV cases matched the predicted pattern of model parameters: purity, copy number, NMD efficiency, as well as relative expression difference between tumor and normal cells. In the assessment with bulk data, we surrogated the *e* as the TPM ratio between tumor and tissue-matched normal controls adjusted for CNAs to evaluate differential expression (Methods) (Fig. 3C). Consistent with our model, *e* was higher for genes carrying elevated AEV indels (median: 1.76 in elevated vs. 0.72 in overall for TSGs; 1.60 vs. 0.92 for nondriver genes; Mann–Whitney *U P* < 2.2 × 10^−16^ for both gene classes). Alternatively, the *e* variable can be greater than one under a global upregulation of transcription activity (hypertranscription), which was recently reported to be widespread in human cancers (Zatzman et al. 2022). Using the degree of hypertranscription in the TCGA cohort by Zatzman et al. (2022) (Methods), we found a higher degree of transcription activity in samples harboring the elevated AEV indels (median: 2.17 in elevated vs.1.77 in overall for TSGs at Mann–Whitney *U P* = 3.1 × 10^−6^; 2.17 vs. 1.63 for nondriver genes at *P* < 2.2 × 10^−16^) (Fig. 3D). AEV is predicted to be elevated by the *e* parameter regardless of mutation clonality (Equation 8 in the Methods) (Supplemental Fig. S3). Thus, we stratified indels by clonality (Methods) and inferred 35.9% of the TCGA indels as subclonal. When clonal and subclonal indels were analyzed separately, their consistent AEV patterns confirmed the robustness of our model to subclonality (Supplemental Fig. S5A). Elevated AEV indels tended to occur in lower-purity samples (median purity: 0.53 in elevated vs. 0.66 in overall for TSGs; 0.49 vs. 0.64 for nondriver genes; Mann–Whitney *U P* < 2.2 × 10^−16^ for both gene classes) (Fig. 3E), which matched the predicted trend in our model. This inverse relationship between elevated AEV and tumor purity was also robust to subclonal mutation (Supplemental Fig. S5B), consistent with the model's projection (Equation 8 in the Methods) (Supplemental Fig. S3). Tumor purity varies greatly by cancer type; for example, tumor samples of pancreatic adenocarcinoma (PAAD) tend to have low purity owing to high stromal content (50%–80%) (Karamitopoulou 2019), whereas those of brain cancers often have high purity (Aran et al. 2016). Indeed, there was a negative correlation between purity and elevated AEV prevalence across the 33 TCGA tumor types (correlation coefficient ρ = –0.67, *P* = 1.9 × 10^−5^) (Fig. 3F), with the highest elevated AEV prevalence (9.38%) detected in PAAD, the cancer type with the lowest tumor purity (median purity of 0.4).

We further examined conditions relevant to truncation indels. Consistent with the model, PTCs caused by elevated AEV indels occurred more frequently in NMD evasive regions (χ^2^ *P* < 2.2 × 10^−16^ for both TSGs and nondriver genes) (Fig. 3G). Because of the higher occurrence of LOH in TSGs (Fig. 3B, right), we used truncation indels in TSGs to examine whether higher allele dosage of NMD-sensitive mutations caused by LOH is accompanied by elevated AEV as projected by our simulation (Fig. 2B; Supplemental Fig. S2). We found that, indeed, LOH was enriched in elevated-AEV indels with NMD efficiency *d* > 0(31.87% vs. 21.19%, Fisher's exact *P* = 1.2 × 10^−3^) (Fig. 3H). In contrast, LOH was not enriched when the truncation indels created PTCs in the last exon, in which the NMD efficiency is close to zero (*d* ≈ 0; 14.43% vs. 14.31%, *P* = 1.0) (Fig. 3H; Lindeboom et al. 2019). Therefore, this result and the higher prevalence of elevated AEV in TSGs (Fig. 3B, left) were consistent with the projection of our model showing an association between LOH and AEV only for *d* > 0 (Fig. 2B; Supplemental Fig. S2).

### Elevated AEV in somatic in-frame indels in TCGA

**Figure 4.**
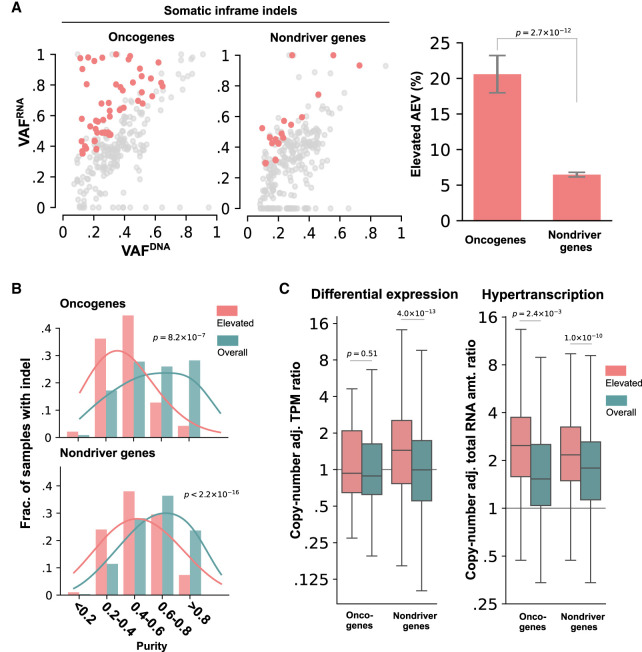
Evaluation of the AEV model using somatic in-frame indels in TCGA samples. Elevated AEV prevalence, comparison of tumor purity, and tumor expression ratio in elevated AEV versus all indels are presented in *A*–*C*, respectively, using the same style and statistical test as the corresponding analyses in Fig. 3. (*A*) Comparison of elevated AEV indels in oncogene and nondriver genes using down-sampled nondriver indels for a scatter plot (*left*) and the full data set (238 indels in oncogenes, 5673 indels in nondrivers) for a bar plot (*right*). (*B*) Comparison of tumor-purity distribution in samples harboring elevated AEV indels (*elevated*) versus all indel-containing samples (*overall*). (*C*) Comparison of TPM ratio based on differential expression (*left*) and hypertranscription (*right*) between *elevated* and *overall* indels.

In-frame indels accounted for 7.07% (7508/106,157) of the TCGA somatic indels and are not expected to trigger NMD. To evaluate the AEV of in-frame indels in driver genes, we focused on oncogenes as in-frame indels accounted for a much higher proportion of somatic indels than in TSGs (41.51% [247/595] vs. 7.86% [403/5127], Fisher's exact *P* < 2.2 × 10^−16^), and included them for analysis if the locus was covered with one or more RNA-seq reads (238 in oncogenes and 5673 in nondrivers) (Supplemental Table S4). Elevated AEV was more frequent in in-frame indels in oncogenes than in nondriver genes (20.59% vs. 6.47%, Fisher's exact *P* = 2.7 × 10^−12^) (Fig. 4A) and was significantly enriched in samples with low tumor purity (median: 0.43 in elevated vs. 0.63 in overall for oncogenes at Mann–Whitney *U P* = 8.2 × 10^−7^) (Fig. 4B). Differential expression between tumor and normal tissues was not a significant contributor to the elevated AEV in oncogenes (median: 0.88 in overall vs. 0.93 in elevated at Mann–Whitney *U P* = 0.51) (Fig. 4C, left), whereas a higher degree of hyperactivation was associated with the elevated pattern (median: 1.52 in overall vs. 2.48 in elevated at *P* = 2.4 × 10^−3^) (Fig. 4C, right). Elevated AEV cases included known gain-of-function in-frame indels such as *EGFR* deletions in exon 19 and insertions in exon 20 (Lynch et al. 2004), *ERBB2* duplications of Y772_A775 (Perera et al. 2009), and *CTNNB1* deletions at S45 (Morin et al. 1997). Considering this, the lack of differential expression may suggest the possibility of bona fide ASE in elevated AEV cases.

### De novo driver indel detection in RNA-seq

**Figure 5.**
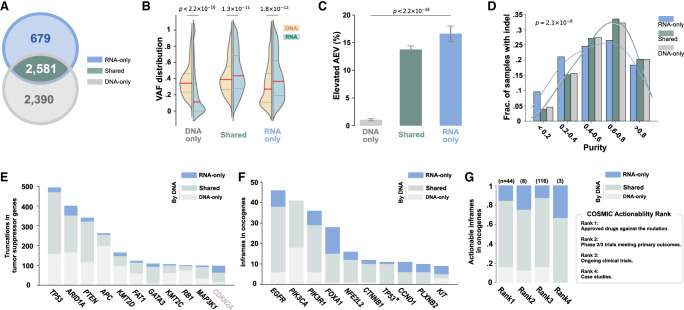
Driver indels detected by RNA-seq and WES in TCGA samples. Driver indels are defined as in-frame mutations in oncogene or truncations in TSGs. (*A*) Venn diagram showing driver indels detected exclusively by GDC's WES analysis (*DNA-only*), by our de novo RNA-seq analysis (*RNA-only*), or by both approaches (*shared*). (*B*) Comparison of VAF distributions in WES (*DNA*) and RNA-seq (*RNA*) by Mann–Whitney *U P*-value for driver indels in these three categories. Median VAF is shown by the red line. (*C*) Comparison of prevalence of elevated AEV indels in these three categories by χ^2^ test. (*D*) Tumor-purity distribution for samples containing indels in the three categories. A Kruskal–Wallis test was performed to assess their differences. (*E*) Top 10 most frequently mutated TSGs contributed by varying indel discovery approaches. *CDKN2A* (ranked top 11th) is also shown as an example for leveraging AEV for discovery owing to its more than 20-fold higher expression in tumors (Supplemental Fig. S7). (*F*) Top 10 most frequently mutated oncogenes. (*) *TP53* was annotated as “possible oncogene” in glioblastoma multiform (GBM) and low-grade glioma (LGG) by Bailey et al. (2018). (*G*) Actionability of oncogenic in-frame indels based on COSMIC Actionability database.

The inverse relationship between elevated AEV and purity (Fig. 2) may be exploited to recover somatic mutations missed by DNA-seq from RNA, especially in low-tumor-purity samples. To test this, we performed a de novo indel detection in TCGA RNA-seq focusing on truncations in TSGs and in-frame indels in oncogenes. A total of 3260 somatic driver indels were called from tumor RNA-seq with one or more DNA supporting reads in the matched WES data (Methods). Compared with 4971 driver indels (4724 truncations in TSGs and 247 in-frame indels in oncogenes) identified from WES data, we found that 2581 were detected by both WES and RNA-seq (Fig. 5A, *shared*), and 679 were detected only by RNAIndel (*RNA-only*) (Supplemental Table S5). The remaining 2390 were detected only by WES (*DNA-only*) owing to low indel allele expression (median allele count: one) with 834 unexpressed.

The increased sensitivity by de novo RNA-seq analysis was not limited to regions with low WES coverage; de novo indel detection by WES plateaued at a coverage of 20×–40× and 40×–60× for TCGA samples with medium-to-high (0.4–1.0) and low (<0.4) purity, respectively (Supplemental Fig. S6), both below the 68× median WES coverage in the TCGA cohort. Although all *RNA-only* indels were validated with WES, matched whole-genome sequencing (WGS) data were available for 476 out of 679 *RNA-only* indels, and 457 of these were also validated by WGS (Supplemental Table S5). For the 19 indels that lacked WGS support, 18 had a high probability (median: 0.37; range: 0.10–1.00) that the absence of indel reads in WGS was owing to insufficient WGS coverage (Methods). The exception event, a hotspot *PIK3R1* in-frame indel in breast cancer (TCGA-LD-A74U), has sufficient WGS coverage, and the absence of indel reads was likely owing to intratumor heterogeneity as the WGS and WES samples were obtained from different vials. The indels in *DNA-only* had a significantly lower VAF^RNA^ than in VAF^DNA^ (median: 0.11 in RNA vs. 0.33 in DNA; Mann–Whitney *U P* < 2.2 × 10^−16^). In contrast, the opposite pattern was found in *RNA-only* indels with much higher VAF^RNA^ (0.36 in RNA vs. 0.26 in DNA, *P* = 1.8 × 10^−12^), and the *shared* indels had a slightly elevated VAF^RNA^ (median: 0.43 in RNA vs. 0.39 in DNA, *P* = 1.3 × 10^−11^) (Fig. 5B). When evaluating elevated AEV, which only occurs when copy-number-adjusted expressions in tumor are higher than in normal, *DNA-only* indels had only a minimum presence (1.1%) in contrast to the significantly high frequency detected in *shared* (13.8%) and *RNA-only* (16.7%, χ^2^ *P* < 2.2 × 10^−16^) (Fig. 5C). Consistent with our model, *RNA-only* indels were enriched in samples with low tumor purity. For example, samples with purity <0.4 accounted for 25.0% *RNA-only* indels in contrast to 14.7% in other categories (Kruskal–Wallis *P* = 2.1 × 10^−8^) (Fig. 5D).

The de novo indels detected by RNA-seq increased truncations in TSGs by 12.7% (n = 602) compared with those known by WES analysis (4724). The top 10 most frequently mutated TSGs had a varying degree of contribution by RNA-seq (Fig. 5E). Although not among the top 10, *CDKN2A* (ranked 11th) is a notable example of having the biggest gain of somatic indels contributed by RNA-seq analysis, resulting in a 35.7% increase owing to the 24.6-fold expression difference in tumor versus normal. In contrast, *RB1* (ranked ninth), which has lower expression in tumor (0.48-fold in tumor), only had a marginal gain (5.0%) by RNA-seq. The differential expression patterns of these two genes in tumor versus normal TCGA bulk samples were also replicated in single-cell RNA-seq (scRNA-seq) data across multiple cancer types (Supplemental Fig. S7). Notably, de novo RNA-seq indel analysis reaped the biggest gain in in-frame indels in oncogenes, with an overall 31.2% increase (77 added to 247 in-frame indels detected by DNA) across multiple oncogenes (Fig. 5F). For example, RNA-seq indels nearly doubled driver indels in *FOXA1*, an oncogene for prostate cancer and breast cancer (Bailey et al. 2018), with 13 RNA indels added to the 15 detected by WES. Of all 28 *FOXA1* indels, the vast majority (71.4%) are located between D249 and E269, which are known to be associated with a favorable prognosis in prostate cancer (Hwang et al. 2025). In addition to prognostic biomarkers, 32.5% (25/77) of the additional oncogene in-frame indels were rated actionable by the COSMIC Actionability database (Fig. 5G; Sondka et al. 2024). These included targetable indels by approved drugs (Rank1 in COSMIC Actionability classification) such as *EGFR* exon 19 deletions in lung adenocarcinoma (n = 5) and *FLT3* deletion at I836 in acute myeloid leukemia (n = 2).

### Single-cell analysis of AEV with subclonal CNA

**Figure 6.**
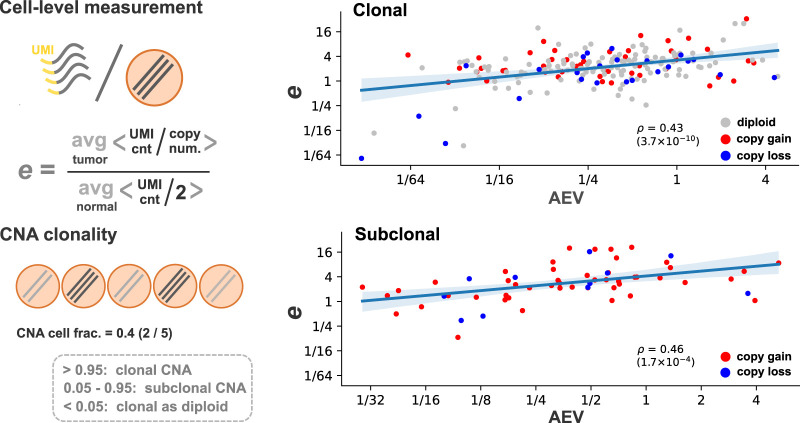
Single-cell model evaluation. Five cutaneous squamous cell carcinoma samples with scRNA-seq and WES were reanalyzed based on the cell type annotation by Ji et al. (2020). In each sample, unique molecular identifier (UMI) counts per gene copy were averaged over cell populations annotated as tumor or normal. The ratio of the tumor average to normal was used as the copy-number-adjusted expression difference between tumor and normal tissues (the *e* parameter). The clonality of CNA events was defined by a fraction of tumor cells carrying the alteration. For substitutions identified from WES, AEV and *e* values were plotted separately for 198 cases in which the background ploidy is clonal and 61 subclonal counterparts (median CNA cell fraction: 0.31).

To validate the AEV model's robustness to subclonal CNA at single-cell level, we analyzed five cutaneous squamous cell carcinomas (cSCC) samples with publicly available scRNA-seq, WES, and cell type annotation (Ji et al. 2020). Specifically, we examined the relationship between AEV and copy-number-adjusted expression difference between tumor and normal cells (the *e* parameter in Equation 8 in the Methods), which is not directly observable in bulk tumor sample. Using scRNA-seq, we derived cell-level gene expression and copy-number estimates accounting for subclonal CNAs (Methods) (Fig. 6). For 223 somatic single-nucleotide and 36 dinucleotide substitutions (e.g., CC > TT) identified from the paired WES with sufficient coverage in scRNA-seq, AEV was determined by the ratio of VAF^RNA^ in a pseudobulk of scRNA-seq to VAF^DNA^ in WES. Consistent with the prediction robustness (Supplemental Fig. S3), higher AEV was correlated with higher *e* values in both clonal (correlation coefficient ρ = 0.43, *P* = 3.7 × 10^−10^) and subclonal (ρ = 0.46, *P* = 1.7 × 10^−4^) events.

## Discussion

In this study, we evaluated the effect of tumor/normal gene expression difference on somatic allele enrichment in bulk RNA-seq. Although a recent study was conceptually aware of such effects (Black et al. 2025), our development of the AEV model has enabled a systematic evaluation of multiple factors contributing to the elevation of AEV. We demonstrated that elevated AEV in a bulk tumor sample can be caused by higher expression in the tumor in absence of ASE. Furthermore, this confounding effect is more pronounced in samples with low tumor purity, which, at first glance, appeared to be paradoxical (Figs. 2A,B, 3E,F). This phenomenon is caused by a slower decline of VAF^RNA^ compared with that of VAF^DNA^ with lower tumor purity when gene expression is higher in the tumor than normal (Fig. 2C). It is noteworthy that the same paradoxical trend can also arise from true ASE, in which the mutant allele is preferentially expressed in the tumor, when examining the enrichment of mutant allele (Supplemental Fig. S8). We demonstrated that this effect could enhance mutation detection from RNA-seq in low-tumor-purity samples (Fig. 5D), whereas DNA-seq was more effective in detecting lowly expressed mutations or those prone to reduced AEV (Fig. 5B,C). In addition to the improved sensitivity by these complementary advantages, integrating DNA-seq and RNA-seq in mutation calling would also reduce false positives by validating putative calls across platforms as demonstrated by the high WGS validation rate for *RNA-only* indels.

Instead of the mutant allele itself, the enrichment of haplotype harboring the mutant allele can be examined using the heterozygous germline SNPs. In general, confounding factors such as tumor/normal expression differences and tumor purity have no or minimum effects on AEV of SNPs (Supplemental Fig. S9). Furthermore, in contrast to AEV of somatic mutation, AEV of SNPs with true ASE would diminish with low tumor purity in the case of true ASE (Supplemental Fig. S8). This SNP-based approach can also be valuable for finding elevated AEV owing to true ASE. For example, when tumor cells are diploid, the VAF^RNA^ of heterozygous SNPs in the bulk tumor sample remains 0.5 if the allele expression is balanced (no ASE), regardless of purity and tumor/normal expression differences (Supplemental Fig. S9A). To illustrate the contrasting patterns, we show allelic imbalance of SNPs associated with *FAT1* V1689fs (AEV: 1.94) in a head-and-neck squamous cell carcinoma, which significantly deviated from 0.5 (Methods) (for simulation-based *P* < 2.2 × 10^−16^, see Supplemental Fig. S10), whereas those with *MAP3K1* P324_N325fs (AEV: 2.23) in breast cancer did not change (*P* = 0.08). When tumor cells are not diploid, SNP VAF^RNA^ also deviates from the expected value defined by CNA with expression differences, but the scale of such deviations is smaller than that in somatic mutations (Supplemental Fig. S9B,C). Thus, SNP-based AEV better controls for the confounders.

A systematic analysis to identify elevated AEV caused by ASE in the current TCGA RNA-seq data is challenging, as the SNP-based method is only applicable to a limited number of cases having a sufficient number of heterozygous exonic SNPs in TCGA RNA-seq data that were prepared with a poly(A) enrichment protocol. Theoretically, VAF^RNA^ is upper bounded by the purity-adjusted VAF^DNA^ (adj. VAF^DNA^; Equation 9 in the Methods) in the absence of ASE. Thus, indels with VAF^RNA^ > adj. VAF^DNA^ may be characterized as true ASE without needing SNPs. When this approach was applied to the TCGA indels (Methods), the estimated ASE prevalence was found to be very low: 0.86% in TSG and 0.19% in nondriver truncating indels and 2.28% in oncogene and 0.37% in nondriver in-frame indels, respectively. Additionally, only highly elevated AEVs were inferred as ASE (average AEV: 3.30 vs. 2.66 for all elevated AEV cases) and moderate cases were missed, including the true ASE event of *FAT1* V1689fs (AEV: 1.94), which was discovered by the SNP-based approach. This limited sensitivity may be because ASE is only tested at a single mutation locus, whereas the SNP-based approach is more statistically powered by testing allele counts at multiple SNP loci.

RNA-seq has been known to be useful for detecting low VAF mutations missed by DNA-based analysis (Xu 2018). Despite this empirical knowledge, it is currently not a common practice to use RNA-seq for mutation calling purposes, possibly because there is no formal theory to demonstrate the advantage of RNA-seq over DNA-seq. Our model projects that RNA-seq can be a more sensitive platform than DNA under the condition that the gene expression per copy is higher in tumor cells than in normal cells, particularly in samples with low tumor purity. Encouragingly, such a condition can be met in a wide range of genes, not restricted to those exhibiting differential expression, owing to a global transcriptional activation in cancers (hypertranscription) (Figs. 4D, 3C; Zatzman et al. 2022). We anticipate that this theoretical basis will motivate future studies to incorporate RNA-seq for mutation detection, especially when reporting therapy-relevant mutations at high sensitivity. For example, in our de novo RNA-seq indel analysis, in-frame indels in oncogenes, which are often actionable, increased by >30% compared with the DNA-based repertoire provided by GDC. Truncation indels can also be important for designing cancer immunotherapy because frameshifts may create potent neoantigens containing amino acid sequences distinct from the self (Turajlic et al. 2017). Among the frameshift indels, only those evading NMD are known to be relevant to therapy (Lindeboom et al. 2019; Litchfield et al. 2020), and it is encouraging that such variants were the most prevalent in indels detected exclusively from RNA-seq (41.5% in *DNA-only* vs. 49.2% in *shared* vs. 63.1% in *RNA-only*, χ^2^ *P* < 2.2 × 10^−16^).

Our current model focuses on elevated AEV as the direction of change is consistent with preferential expression of mutant alleles, although AEV can be elevated (VAF^RNA^ > VAF^DNA^) or reduced (VAF^RNA^ < VAF^DNA^). Reduced AEV of truncation indels was less prevalent in TSGs than in the nondrivers (9.15% vs. 12.76%, Fisher's exact *P* = 2.5 × 10^−13^) (Supplemental Fig. S11A), and as expected, such indels are mostly located in the NMD-sensitive regions in both gene categories (Supplemental Fig. S11B). The difference can be attributed to higher LOH frequency in TSGs (Fig. 3B, right), which results in higher mutant allele dosage. Our model projects that AEV is higher with enriched mutant allele dosage in the presence of NMD (Fig. 2B). Therefore, the lower prevalence of reduced AEV in TSGs further supports the opposing effect of NMD and LOH proposed by our model. Consistent with the absence of NMD in in-frame indels, reduced AEV in these variants was rare in both oncogenes (2.79%) and nondrivers (4.08%); Fisher's exact *P* = 0.41) (Supplemental Fig. S11C).

In conclusion, we identified tumor/normal expression difference as a confounding factor contributing to enriched somatic mutant allele expression in bulk tumor samples. The confounding effect is particularly pronounced in low-purity tumor samples. These findings serve to caution the interpretation of allelic imbalance characterized by somatic mutation alone, which suggests the inclusion of germline heterozygous SNPs for detecting bona fide ASE. A comprehensive assessment of ASE of somatic mutation may need to incorporate noncoding *cis*-regulatory variants from WGS, DNA methylation, and miRNA-seq data along with RNA-seq data generated from total RNA extraction to capture more heterozygous SNPs. Of note, this study yielded a valuable byproduct: The confounding effect can be exploited to efficiently complement DNA-based mutation detection in samples with low tumor purity by calling somatic mutation from RNA-seq.

## Methods

### TCGA data set

This study used the TCGA data set available as GDC v36 on the CGC (https://www.cancergenomicscloud.org). We included TCGA cases for which tumor/normal-paired WES and tumor RNA-seq BAM files and mutation annotation format (MAF) files (n = 9101) were all available. The 9101 data pairs of tumor WES and RNA-seq were from all primary tumor samples, and tumor DNA and RNA were extracted from the same vial for 9058 samples (99.5%). The remaining 43 cases using different vials for DNA/RNA extraction were all acute myeloid leukemia, a liquid tumor, that are not expected to have extensive spatial heterogeneity. The MAF files were prepared by GDC by applying MuTect2 (Cibulskis et al. 2013), VarScan2 (Koboldt et al. 2012), and Pindel (Ye et al. 2009) to the paired WES for somatic indel calls. This multicaller pipeline does not require concordance across callers to generate the somatic indel data set (Zhang et al. 2021). As a result, 61.0% of the somatic indels were detected by all three callers; 1.4% were detected by a single caller, with the remaining 37.6% by two callers. Gene expression data by STAR Count (Dobin et al. 2013) and copy-number data by ASCAT (Van Loo et al. 2010) were also available on the CGC. The ABSOLUTE-based tumor-purity data for TCGA samples was obtained from TCGA PanCanAtlas (https://gdc.cancer.gov/about-data/publications/pancanatlas). The platform to generate this purity data was SNP6, and tumor samples using the same vial for SNP6, WES, and RNA-seq accounted for 8481 of 9101 cases. To ensure analytical consistency, we excluded 620 cases with unmatched vials from any analysis that involved the tumor-purity data, and their purity value was set to “–1” in Supplemental Tables S3–S5 to indicate missing values.

### Derivation of VAF ratio between RNA and DNA

In a bulk tumor sample consisting of *x* tumor and *y* normal cells, we denote *q* as

q=y/x=(1−purity)/purity,wherepurity=x/(x+y).

For an autosomal gene harboring a somatic mutation in tumor cells, we expect *m* and *w* copies of mutant and wild-type alleles per cell, respectively. *m* is a positive integer, and *w* is a nonnegative integer, where *w* = 0 represents loss of the wild-type allele owing to LOH. The normal cells are diploid and homozygous for the reference allele. The DNA VAF in the bulk tumor sample is given by

(1) $$VAF^{DNA}=mx/(mx+wx+2y)=m/(m+w+2q).$$

Consider that each gene copy in tumor cells is expressed at *e*-fold relative to normal, regardless of the mutation status of the allele (i.e., no ASE). The mutant allele triggers NMD with decay efficiency *d* ∈ [0, 1], where *d* = 0 represents a complete evasion from NMD, and *d* = 1 represents a complete decay of the transcript. The RNA VAF is

(2) $$\begin{array}{rl} VAF^{RNA} & =e\cdot mx\cdot (1-d)/\{e\cdot mx\cdot (1-d)+e\cdot wx+2y\}\\ & =e\cdot m\cdot (1-d)/\{e\cdot (m+w)-e\cdot m\cdot d+2q\} \end{array}.$$

The VAF ratio of RNA to DNA (i.e., AEV) is

(3) $$VAF^{RNA}/VAF^{DNA}=\;e\cdot (1-d)\cdot (m+w+2q)/\{e\cdot (m+w)-e\cdot m\cdot d+2q\}.$$

When the VAF ratio is greater than one, solving for *e*, we have

e⋅{2q⋅(1−d)−w⋅d}>2q.

Because *e* and *q* are positive, it follows that 2*q* · (1 − *d*) − *w* · *d* > 0. As *w* and *d* are nonnegative, *e* must stratify the following for elevated AEV:

(4) e>2q/(2q−2q⋅d−w⋅d)>1.

In the absence of NMD (i.e., *d* = 0), Equation 3 becomes

(5) $$\begin{array}{rl} VAF^{RNA}/VAF^{DNA} & =\;e\cdot (m+w+2q)/\{e\cdot (m+w)+2q\}\\ & =e\cdot (c+2q)/(e\cdot c+2q),\;\;\;\mathrm{where}\;\;c=m+w\;. \end{array}$$

Thus, the ratio depends on the copy number *c* but not the genotype. For example, when the copy number in the tumor cells is three (i.e., *c* = 3), AEV is identical in all genotypes: *m*:*w* = 1:2, 2:1, or 3:0. In the presence of NMD, solving Equation 4 for *d*, we have the following as the efficiency upper bound for elevated AEV:

(6) d<2q/(2q+w)⋅(e−1)/e≤(e−1)/e.

CNA in bulk tumor samples may be subclonal. This can be modeled by introducing a new parameter, *i* ∈ [1, 0], which represents *i* · *x* of tumor cells harboring the CNA event. Somatic mutations may also be subclonal, and their occurrence with CNA may be independent or may be associated as seen in the case of deactivating TSG mutations with LOH. To capture any scenarios, the mutation subclonality is separately parameterized for tumor cells with and without CNA by *j* ∈ [0, 1] and *k* ∈ [0, 1], where the mutation is found in *i* · *j* · *x* tumor cells with CNA and (1 − *i*) · *k* · *x* those without. Under this formulation, the subclonal DNA VAF is

(7) $$VAF^{DNA}=\{i\cdot j\cdot m+(1-i)\cdot k\}/\{(c-2)\cdot i+(1+q)\cdot 2\}.$$

The subclonal RNA VAF is

$$VAF^{RNA}=e\cdot \{i\cdot j\cdot m+(1-i)\cdot k\}\cdot (1-d)/\{e\cdot i\cdot (c-m\cdot d\cdot j)+e\cdot (1-i)\cdot (2-d\cdot k)+2q\}.$$

The AEV model for subclonal case is derived as

(8) e⋅{(c−2)⋅i+(1+q)⋅2}⋅(1−d)/{e⋅i⋅(c−m⋅d⋅j)+e⋅(1−i)⋅(2−d⋅k)+2q}.

The model was studied by simulation with a custom script (*aev_sim.py*).

### Indel allele count benchmarking

The Sequencing Quality Control Phase 2 (SEQC2) consortium established a reference somatic call set from the HCC1395 breast cancer cell line and the HCC1395BL cell line, which was derived from the normal B cell of the same donor (Fang et al. 2021). We downloaded the reference indel call set (https://ftp-trace.ncbi.nlm.nih.gov/ReferenceSamples/seqc/Somatic_Mutation_WG/release/v1.2.1/). The consortium also generated WGS data on a series of diluted samples by mixing *i* parts of DNA material from HCC1395 and *j* parts from HCC1395BL for *i*:*j* = 1:0 (undiluted), 3:1, 1:1, 1:4, 1:9, and 1:19 (Zhao et al. 2021; https://ftp-trace.ncbi.nlm.nih.gov/ReferenceSamples/seqc/Somatic_Mutation_WG/data/SPP/). The true VAF of somatic mutations is unknown. Therefore, we used the dilution ratio as the ground-truth value. Specifically, the following equation holds for the amount of mutated DNA material between a sample at *n*:*m* and the undiluted sample:

$$k\times VAF^{i:j}=\;\{\;k\times i/(i+j)\}\times VAF^{1:0},\;\;\mathrm{where}\;k\;\;\mathrm{is}\;\mathrm{the}\;\mathrm{total}\;\mathrm{DNA}\;\mathrm{amount}.$$

With this relation, the dilution ratio ideally matches the observed VAF ratio:

$$\;VAF_{x\;}^{i:j}/VAF_{x\;}^{1:0}=\;i/(i+j),\;\mathrm{for}\;\mathrm{tool}\;x.$$

Using the serial dilution WGS data set, we compared the variant callers used in the GDC pipeline (Zhang et al. 2021) against indelPost (v0.2.3) (Hagiwara et al. 2022) for the dilution ratio estimation. For VarScan2 (v2.3.9) (Koboldt et al. 2012), we used the *pileup2indel* subcommand with *‐‐min-coverage 1 ‐‐min-reads2 1* and *‐‐min-var-freq 0.0001* to increase sensitivity. Pindel (v0.2.4) (Ye et al. 2009) was used with *-insert-size 400* as estimated (Zhao et al. 2021). MuTect2 (v4.1.8.0) (Cibulskis et al. 2013) was used with the default settings. indelPost's *count_alleles()* function was applied with the *by_fragment* option, which merges forward and reverse reads if they are from the same fragment. At 1:0, indelPost detected all the high-confidence indels (n = 1467), whereas 1445 were found by MuTect2, 1196 by VarScan2, and 1432 by Pindel. We used the unfiltered outputs from these callers (MuTect2, VarScan2, and Pindel) to maintain their sensitivity. Compared with these tools, indelPost consistently generated the best match to the expected ratios of 3/4, 1/2, 1/5, 1/10, and 1/20 over the dilution series (Supplemental Fig. S4A; Supplemental Table S1).

In RNA-seq, the VAF from the diluted samples generally does not match the dilution ratio owing to gene expression differences between the tumor and normal cells. Therefore, we used an RNA-seq data for the Genome in a Bottle (GIAB) reference germline indels in the NA12878 lymphoblastoid cell line (Zook et al. 2014; obtained from the NCBI BioProject database [https://www.ncbi.nlm.nih.gov/bioproject/] under accession number PRJNA389940) to evaluate the consistency of indel VAF in RNA-seq with their genotype in DNA. We first selected expressed exonic indels covered with one or more RNA-seq reads in the autosomal chromosomes. To avoid bias from NMD, we retained indels annotated as in-frame or as in untranslated regions (UTRs) if there were no co-occurring nonsense SNPs, frameshifts, or variants affecting splicing in the target gene. To avoid bias from ASE, we excluded genes identified to have ASE based on a prior ASE study that included NA12878 (Chen et al. 2016). Consequently, the indels retained for this analysis are expected to exhibit balanced biallelic expressions with VAF^RNA^ values of 0.5 and 1.0 for the heterozygous and homozygous genotypes, respectively. The indel allele count tools were run with the same parameter settings as in the DNA benchmarking. The VAF^RNA^ estimates by indelPost were the closest to the expected heterozygous and homozygous genotypes of NA12878, which were determined by a pedigree analysis by the GIAB consortium (Supplemental Fig. S4B; Supplemental Table S2).

### Indel annotation

Indels were annotated for amino acid changes by VEP (v100) (McLaren et al. 2016). Those annotated as frameshift or in-frame insertion of a stop codon were collectively defined as truncation indels, whereas the other in-frame indels were defined as in-frame indels. Bailey et al. (2018) classified genes as drivers if their mutation frequencies were significant in the entire TCGA data set (i.e., pancancer drivers) or in individual TCGA tumor types (i.e., cancer-specific drivers). Using this resource, we annotated genes as drivers, if the model predicted the gene to be a pancancer driver, or as a cancer-specific driver in the sample's tumor type. Microsatellite instability (MSI) can induce mutations in driver genes regardless of their functional consequences, leading to overannotation of driver status. To mitigate this confounding factor, we only used cancer-specific driver genes in MSI-enriched tumor types (COAD, ESCA, STAD, and UCEC). Indels were annotated as drivers if they occurred in driver genes. Bailey et al. (2018) also provide tumor suppressor/oncogene classification in two confidence levels: “*tsg*” or “*possible tsg*” and “*oncogene*” or “*possible oncogene.*” In the current study, these levels were combined to annotate driver indels of either TSGs or oncogenes.

### AEV analysis

Empirically, AEV is estimated from allele count, a summary statistic contributed by all the parameters in the AEV equation (Eqs. 3, 8). To ensure accurate VAF estimation in RNA-seq and WES, read counts for somatic indels in the TCGA data set (Fig. 1, *DNA somatic indels*) were analyzed by a custom script run on CGC (*cross_platform_checker.py*), in which indelPost (Hagiwara et al. 2022) was used for realignment. This process also harmonizes the inconsistency in indel representations across the variant callers used by GDC or correct inaccurate allele reports (Supplemental Fig. S12). The mutant and wild-type allele counts in tumor WES and RNA-seq were compared by Fisher's exact test and were considered significantly different if the *P*-value was <0.01 after Benjamini–Hochberg correction (Benjamini and Hochberg 1995). The effect size threshold was set at 20%, which required VAF^RNA^/VAF^DNA^ to be greater than 1.2 for elevated AEV and less than 0.8 for reduced AEV.

To test if the model is robust to subclonal mutations, we characterized the clonality of somatic indels based on the allele-specific copy-number estimate by ASCAT (see the section “TCGA data set”). For genomic intervals, the copy number was separately estimated as major and minor alleles, where *major_copy_num* ≥ *minor_copy_num*. The TCGA indels were annotated with the ASCAT results based on the genomic interval overlap. Assuming that CNAs detected by ASCAT were primarily clonal, the subclonal VAF^DNA^ equation (Equation 7) with the CNA clonality parameter *i* = 1 becomes

$$\;VAF^{DNA}\;\times (c+2q)\;=\;j\cdot m,\;\;\mathrm{where}\;\;q=(1-\mathrm{purity})/\mathrm{purity}.$$

Because the number of mutant allele copies (i.e., *m*) is equal to or less than *major_copy_num*, the cancer cell fraction with mutation (i.e., *j*) has a lower-bound estimate:

$$VAF^{DNA}\;\times (c+2q)\;/\;\mathrm{major\_copy\_num}\;\leq j.$$

Clonal indels are expected to have *j* = 1 and *j* < 1 would be observed for subclonal. Thus, indels were defined as clonal if the one-sided 95% confidence interval contains/exceeds one and are subclonal otherwise.

Genomic intervals with *minor_copy_num* = 0 were regions with LOH because one of the diploid alleles was lost, and indels in these regions were potentially coupled with LOH. For such indels, we adjusted the raw VAF^DNA^ value with purity and copy number to ensure the loss of the wild-type allele:

(9) $$\mathrm{adj}.VAF^{DNA}=\;VAF^{DNA}\;\times \;(c+2q)/c=m/c.$$

We used those with the adjusted VAF^DNA^ > 0.9 to analyze the impact of NMD on AEV.

### NMD analysis

Two related studies by Lindeboom et al. (2016, 2019) estimated NMD efficiency by comparing expression levels of various truncated transcripts to the wild-type transcript and proposed a NMD rule. By this rule, an mRNA transcript containing a PTC is classified as NMD evasive if the PTC position is <150 nt downstream from the start codon (estimated NMD efficiency = 0.12), is in an exon spanning >407 nt (0.41), is <55 nt upstream of the last exon junction (0.20), or is within the last exon (∼0); otherwise, the PTC is considered sensitive to NMD (0.65). In this measurement, an NMD efficiency of 0.2 would result in a 10% expression reduction in a sample with a heterozygous truncating mutation in a diploid region. In our model, the NMD efficiency parameter *d* also simulates this expression change: a 10% reduction of the number of transcripts per cell at *d* = 0.2 with *m* = 1 and *w* = 1. For each frameshift, we searched for the first downstream occurrence of a PTC along RefSeq isoforms (Pruitt et al. 2005) using a custom script (*premature_stop_codon_locator.py*) and annotated the frameshifts for the NMD features based on the PTC position.

### Relative expression difference surrogation

The tumor versus normal expression ratio per gene copy (variable *e* in the AEV model) was surrogated in a bulk sample under two scenarios: differential gene expression and hypertranscription. For differential expressions, we first divided the gene's transcripts per million (TPM) in the tumor sample by its copy number as estimated by ASCAT. As the autosomal chromosomes in normal cells are diploid, we halved the median TPM from tissue-matched normal expression data also taken from TCGA for technical consistency. We used the copy-number-adjusted ratio of TPM in tumor to normal median as an estimate for differential expression. Cancer types with fewer than five normal matching RNA-seq data were excluded in this analysis. Zatzman et al. (2022) estimated the degree of hypertranscription at sample level as a ratio of total RNA amount in tumor divided by that in normal with ploidy adjusted, and we used their estimates for the TCGA data set.

### Single-cell data analysis

A publicly available scRNA-seq data set was downloaded for cSCC samples along with their matched tumor/normal-paired WES and cell type annotation with tumor cells (n = 5; obtained from the NCBI Gene Expression Omnibus [https://www.ncbi.nlm.nih.gov/geo/] under accession number GSE144240) (Ji et al. 2020). The scRNA-seq reads were mapped to GRCh38 by 10x Genomics’ Cell Ranger (v8.0.1), which also generated a unique molecular identifier (UMI) count matrix with each gene in each cell. We used inferCNV (v1.18.1) to estimate gene copy number in each tumor cell using the cell type annotations as reported by the original study. In a tumor cell barcoded by *t*, the copy-number-adjusted expression of gene X is estimated by dividing the count of gene X's UMIs in the cell ($UMI_{t}^{X}$) by the copy number of gene X in the cell ($c_{t}^{X}$). In the original study, Ji et al. (2020) identified tumor basal, cycling, and differentiating keratinocytes and tumor-specific keratinocyte as cell types uniquely localized to the tumor lesions by a spatial transcriptomic analysis. We used the set of cell barcodes annotated for these cell types (*T*) to represent the tumor cell population, whereas the normal cell population was represented by the remaining set of barcodes (*N*). Assuming that normal cells were diploid, the tumor/normal expression difference per copy in a given sample was estimated for gene X as

$$\begin{array}{r} \frac{\left|N\right|}{\left|T\right|}\times \frac{\sum_{t\;\in \;T}UMI_{t}^{X}/c_{t}^{X}}{\sum_{n\;\in \;N}UMI_{n}^{X}/2},\;\;\mathrm{where}\;|\;\;|\;\mathrm{denotes}\;\mathrm{the}\;\mathrm{size}\;\mathrm{of}\;\mathrm{barcode}\;\mathrm{set}. \end{array}$$

The fraction of tumor cells with CNA at gene X was calculated based on the cell-level copy-number estimate at gene X :

$$\mathrm{Frac}.\;\mathrm{of}\;\mathrm{tumor}\;\mathrm{cells}\;\mathrm{with}\;\;CNA=\;|\left\{t|c_{t}^{X}\neq 2\;for\;t\in T\right\}|/|T|.$$

We defined the CNA clonality by this fraction: clonal CNA if greater than 0.95, diploid if less than 0.05, and subclonal CNA otherwise. After mapping the downloaded WES reads to GRCh38 by BWA (v 0.7.12-r1039), MuTect2 (v4.2.6.1) was applied to the paired WES data using the default settings. Somatic mutations on autosomal chromosomes, which passed *FilterMutectCalls* at VAF^DNA^ > 0.05, were annotated by VEP (v100) to retain those in gene regions. Allele counts were derived from the alignment pileup of scRNA-seq reads, where the coverage was required of five or more with two or more mutant supporting reads, to estimate a pseudobulk VAF^RNA^ and to estimate AEV by dividing with the VAF^DNA^.

To analyze *CDKN2A* and *RB1* expression, single-cell/single-nucleus RNA-seq (sc/snRNA-seq data) and cell type annotations were obtained from the previous study (Terekhanova et al. 2023). The methods for data normalization, feature selection, dimensionality reduction, and data merging were the same as used in that study. Average gene expression visualization in the selected cell groups was performed using DotPlot function from the Seurat package (v5.1.0) (Butler et al. 2018).

### Somatic indel calling from RNA-seq

De novo indel calling was carried out by running RNAIndel (Hagiwara et al. 2020) on CGC. Computing cost was a key consideration on this platform, so we refactored RNAIndel by replacing the original realignment module with the much more efficient indelPost. This refactored version ran about 10 times faster (median run time: 21 vs. 208 min/sample) and achieved an ∼85% reduction in computing cost (median cost: 0.16 vs. 0.97 USD/sample) (Supplemental Table S6). We deployed this code (v.3.3.0) to CGC for the full analysis, and the cost ranged from 0.04 to 14.8 dollars/sample (median: 0.21). On CGC, RNAIndel (v.3.3.0) analysis on tumor RNA-seq BAM files used the default parameters except for the parallelism option, which was set to 12. In the output VCF files, putative indels were classified as somatic, germline, or artifactual based on the machine-learning model implemented in RNAIndel. Those classified as somatic were extracted from the VCF file and annotated for driver status (*driver annotation*).

For each sample, all somatic driver indels by RNAIndel and those by the GDC pipeline were realigned on the corresponding paired WES data by indelPost imported in a custom script (*cross_platform_checker.py*) to harmonize the indel representation ambiguity across callers and/or platforms (Supplemental Fig. S12). Indels called by GDC were defined as *DNA-only* (Fig. 5A) if they did not match any of indels called from RNA after harmonization within the sample and as *shared* if they matched. Using the read count from the realignment process, we validated harmonized indels unique to RNA by testing for the presence of mutant reads in the tumor WES and their absence in the normal data or by testing for a significantly higher VAF in tumor by proportion test if mutant reads were present in the normal data. WES-validated RNA findings (*RNA-only*) were further scrutinized by a tumor/normal-paired WGS data set if available. We included the WGS data at the sample-match level to maximize the data set size available for similar validation. For indels not validated by WGS, we calculated the probability that a mutation present at the VAF^DNA^ estimated in the tumor WES was not captured in the tumor WGS coverage using the binomial model: (1 − *VAF*^*DNA*^)^*WGS* coverage^.

To evaluate the sensitivity of de novo indel detection in the TCGA WES data set at varying coverages and tumor purities, we used the somatic indel set composed of *DNA-only*, *shared*, and *RNA-only* indels (Fig. 5A). The somatic origin of *RNA-only* indels were validated as described above. Samples were stratified into three categories of low (0.1–0.4), medium (0.4–0.7), and high (0.7–1.0) tumor purity. The sensitivity of de novo indel detection in WES was defined as the proportion of *DNA-only* and *shared* indels out of all indels detected in each of coverage bins: 0×–20×, 20×–40×, 40×–60×, 60×–80×, 80×–100×, and >100×.

### Actionability annotation

We focused on in-frame indels in oncogenes using the COSMIC Actionability database (v14) (Sondka et al. 2024) by querying gene name and tumor type for the fields of GENE and DISEASE in the database. If a match was found, we examined if the mutation curated in the MUTATION_REMARK field matched the input in-frame indel. If a curated mutation was not specified, we considered the in-frame indel a match. For the matched in-frame indels, we retrieved the actionability category curated in the ACTIONABILITY_RANK field: Rank1, approved drug demonstrated efficacy at the mutation; Rank2, phase2/3 clinical results meeting primary outcomes; Rank3, drug in ongoing clinical trials; and Rank4, case studies only.

### Assessment of true ASE events

We adopted a simulation-based *P*-value computation developed for somatic silencing of the *BARD1* gene in neuroblastoma (Cupit-Link et al. 2024). Briefly, using heterozygous SNPs annotated as common by dbSNP (Sherry et al. 2001), we calculated the sum of deviation from the heterozygous VAF (i.e., 0.5) in RNA-seq:

$$\underset{SNPs}{\sum }|VAF^{RNA}-0.5|=\mathrm{observed}\;\mathrm{total}\;\mathrm{deviation}.$$

We simulated the total deviation by a binomial model with RNA-seq coverage at each SNP locus:

$$\underset{SNPs}{\sum }|\mathrm{binom}(\mathrm{cov},\;0.5)/\mathrm{cov}-0.5|,\;\;\mathrm{where}\;\mathrm{cov}\;\mathrm{is}\;\mathrm{RNA}\;\mathrm{coverage}.$$

We counted the number of simulations deviating more than the observed value. The *P*-value was calculated by dividing the count by the total number of simulations (n = 1000).

Putative ASE events were also assessed without using SNPs. In the absence of ASE, VAF^RNA^ (Equation 2) is upper-bounded by the purity-adjusted VAF^DNA^ (Equation 9):

$$\underset{e\to \infty }{\lim}VAF^{RNA}=m/\{m+w/(1-d)\}\;\leq \;m/(m+w)=m/c,\;\;\mathrm{where}\;c=m+w.$$

Assuming that the mutant allele is from tumor, the count of wild-type allele from tumor is adjusted from the total wild-type allele count:

$$\mathrm{adj}.\;\mathrm{wt}\;\mathrm{allele}\;\mathrm{cnt}=\;(\mathrm{total}-\mathrm{total}\cdot \mathrm{adj}\;VAF^{DNA})/\mathrm{adj}\;VAF^{DNA}.$$

In the actual computation, we capped adj. VAF^DNA^ at 1.0, and the adjusted allele count was floored to integer. Similar to the AEV analysis, the mutant and adjusted wild-type allele count in WES was compared to the raw RNA-seq allele count by a Fisher's exact test with Benjamini–Hochberg correction. Indels with VAF^RNA^ > adj. VAF^DNA^ at FDR < 0.01 were defined as ASE events.

### Statistical analysis

For count data, Fisher's exact and χ^2^ tests were used for pairwise and three-group comparisons, respectively. To compare distributions, Mann–Whitney *U* and Kruskal–Wallis tests were used for pairwise and three-group comparisons. All tests were two-sided and were taken significant at *P*-value < 0.05 unless otherwise specified.

### Code availability

Custom codes developed for this study are available at Zenodo (https://zenodo.org/records/17602849) and as Supplemental Code. The refactored version of RNAIndel has been integrated into the original code repository as version 3 (https://github.com/stjude/RNAIndel), and the v3.3.0 code, which was used in the current study, is also included in Supplemental Code.

## Supplemental Material

Supplement 1

Supplement 2

Supplement 3

Supplement 4

Supplement 5

Supplement 6

Supplement 7

Supplement 8

Supplement 9

Supplement 10

Supplement 11

Supplement 12

Supplement 13

Supplement 14

Supplement 15

Supplement 16

Supplement 17

Supplement 18

Supplement 19

## References

[GR281003HAGC1] Alioto TS, Buchhalter I, Derdak S, Hutter B, Eldridge MD, Hovig E, Heisler LE, Beck TA, Simpson JT, Tonon L, 2015. A comprehensive assessment of somatic mutation detection in cancer using whole-genome sequencing. Nat Commun 6: 10001. 10.1038/ncomms10001 26647970 PMC4682041

[GR281003HAGC2] Aran D, Sirota M, Butte AJ. 2016. Systematic pan-cancer analysis of tumour purity. Nat Commun 6: 8971. 10.1038/ncomms9971 PMC4671203 26634437

[GR281003HAGC3] Bailey MH, Tokheim C, Porta-Pardo E, Sengupta S, Bertrand D, Weerasinghe A, Colaprico A, Wendl MC, Kim J, Reardon B, 2018. Comprehensive characterization of cancer driver genes and mutations. Cell 174: 1034–1035. 10.1016/j.cell.2018.07.034 30096302 PMC8045146

[GR281003HAGC4] Benjamini Y, Hochberg Y. 1995. Controlling the false discovery rate: a practical and powerful approach to multiple testing. J Royal Stat Soc Ser B 57: 289–300. 10.1111/j.2517-6161.1995.tb02031.x

[GR281003HAGC5] Black JRM, Jones TP, Martínez-Ruiz C, Litovchenko M, Puttick C, Swanton C, McGranahan N. 2025. Cancer gene identification from RNA variant allelic frequencies using RVdriver. Genome Biol 26: 165. 10.1186/s13059-025-03557-y 40514689 PMC12164115

[GR281003HAGC6] Bollas A, Gaither J, Schieffer KM, White P, Mardis ER. 2025. Variant calling from RNA-seq data reveals allele-specific differential expression of pathogenic cancer variants. Commun Med 5: 202. 10.1038/s43856-025-00901-y 40437029 PMC12119874

[GR281003HAGC7] Bowry A, Kelly RDW, Petermann E. 2021. Hypertranscription and replication stress in cancer. Trends Cancer 7: 863–877. 10.1016/j.trecan.2021.04.006 34052137

[GR281003HAGC8] Butler A, Hoffman P, Smibert P, Papalexi E, Satija R. 2018. Integrating single-cell transcriptomic data across different conditions, technologies, and species. Nat Biotechnol 36: 411–420. 10.1038/nbt.4096 29608179 PMC6700744

[GR281003HAGC9] Cancer Genome Atlas Research Network. 2013. The Cancer Genome Atlas Pan-Cancer analysis project. Nat Genet 45: 1113–1120. 10.1038/ng.2764 24071849 PMC3919969

[GR281003HAGC10] Castel SE, Levy-Moonshine A, Mohammadi P, Banks E, Lappalainen T. 2015. Tools and best practices for data processing in allelic expression analysis. Genome Biol 16: 195. 10.1186/s13059-015-0762-6 26381377 PMC4574606

[GR281003HAGC11] Chen J, Rozowsky J, Galeev TR, Harmanci A, Kitchen R, Bedford J, Abyzov A, Kong Y, Regan L, Gerstein M. 2016. A uniform survey of allele-specific binding and expression over 1000-Genomes-Project individuals. Nat Commun 7: 11101. 10.1038/ncomms11101 27089393 PMC4837449

[GR281003HAGC12] Chen Z, Guo Y, Zhao D, Zou Q, Yu F, Zhang L, Xu L. 2021. Comprehensive analysis revealed that *CDKN2A* is a biomarker for immune infiltrates in multiple cancers. Front Cell Dev Biol 9: 808208. 10.3389/fcell.2021.808208 35004697 PMC8733648

[GR281003HAGC13] Cibulskis K, Lawrence MS, Carter SL, Sivachenko A, Jaffe D, Sougnez C, Gabriel S, Meyerson M, Lander ES, Getz G. 2013. Sensitive detection of somatic point mutations in impure and heterogeneous cancer samples. Nat Biotechnol 31: 213–219. 10.1038/nbt.2514 23396013 PMC3833702

[GR281003HAGC14] Cupit-Link M, Hagiwara K, Nagy M, Koo SC, Orr BA, Ruppin E, Easton J, Zhang J, Federico SM. 2024. Response to PARP inhibition in *BARD1*-mutated refractory neuroblastoma. N Engl J Med 391: 659–661. 10.1056/NEJMc2403316 39141861 PMC11328958

[GR281003HAGC15] Dobin A, Davis CA, Schlesinger F, Drenkow J, Zaleski C, Jha S, Batut P, Chaisson M, Gingeras TR. 2013. STAR: ultrafast universal RNA-seq aligner. Bioinformatics 29: 15–21. 10.1093/bioinformatics/bts635 23104886 PMC3530905

[GR281003HAGC16] Fang LT, Zhu B, Zhao Y, Chen W, Yang Z, Kerrigan L, Langenbach K, de Mars M, Lu C, Idler K, 2021. Establishing community reference samples, data and call sets for benchmarking cancer mutation detection using whole-genome sequencing. Nat Biotechnol 39: 1151–1160. 10.1038/s41587-021-00993-6 34504347 PMC8532138

[GR281003HAGC17] Ge B, Pokholok DK, Kwan T, Grundberg E, Morcos L, Verlaan DJ, Le J, Koka V, Lam KC, Gagné V, 2009. Global patterns of *cis* variation in human cells revealed by high-density allelic expression analysis. Nat Genet 41: 1216–1222. 10.1038/ng.473 19838192

[GR281003HAGC18] Hagiwara K, Ding L, Edmonson MN, Rice SV, Newman S, Easton J, Dai J, Meshinchi S, Ries RE, Rusch M, 2020. RNAIndel: discovering somatic coding indels from tumor RNA-seq data. Bioinformatics 36: 1382–1390. 10.1093/bioinformatics/btz753 31593214 PMC7523641

[GR281003HAGC19] Hagiwara K, Edmonson MN, Wheeler DA, Zhang J. 2022. indelPost: harmonizing ambiguities in simple and complex indel alignments. Bioinformatics 38: 549–551. 10.1093/bioinformatics/btab601 34431982

[GR281003HAGC20] Hwang J, Likasitwatanakul P, Deshmukh SK, Wu S, Kwon JJ, Toye E, Moline D, Evans MG, Elliott A, Passow R, 2025. Structurally oriented classification of *FOXA1* alterations identifies prostate cancers with opposing clinical outcomes and distinct molecular and immunologic subtypes. Clin Cancer Res 31: 936–948. 10.1158/1078-0432.CCR-24-3471 39745364 PMC11873805

[GR281003HAGC21] The ICGC/TCGA Pan-Cancer Analysis of Whole Genomes Consortium. 2020. Pan-cancer analysis of whole genomes. Nature 578: 82–93. 10.1038/s41586-020-1969-6 32025007 PMC7025898

[GR281003HAGC22] Ji AL, Rubin AJ, Thrane K, Jiang S, Reynolds DL, Meyers RM, Guo MG, George BM, Mollbrink A, Bergenstråhle J, 2020. Multimodal analysis of composition and spatial architecture in human squamous cell carcinoma. Cell 182: 497–514.e22. 10.1016/j.cell.2020.05.039 32579974 PMC7391009

[GR281003HAGC23] Karamitopoulou E. 2019. Tumour microenvironment of pancreatic cancer: immune landscape is dictated by molecular and histopathological features. Br J Cancer 121: 5–14. 10.1038/s41416-019-0479-5 31110329 PMC6738327

[GR281003HAGC24] Knudson AG Jr. 1971. Mutation and cancer: statistical study of retinoblastoma. Proc Natl Acad Sci 68: 820–823. 10.1073/pnas.68.4.820 5279523 PMC389051

[GR281003HAGC25] Koboldt DC, Zhang Q, Larson DE, Shen D, McLellan MD, Lin L, Miller CA, Mardis ER, Ding L, Wilson RK. 2012. VarScan 2: somatic mutation and copy number alteration discovery in cancer by exome sequencing. Genome Res 22: 568–576. 10.1101/gr.129684.111 22300766 PMC3290792

[GR281003HAGC26] Lau JW, Lehnert E, Sethi A, Malhotra R, Kaushik G, Onder Z, Groves-Kirkby N, Mihajlovic A, DiGiovanna J, Srdic M, 2017. The Cancer Genomics Cloud: collaborative, reproducible, and democratized-A new paradigm in large-scale computational research. Cancer Res 77: e3–e6. 10.1158/0008-5472.CAN-17-0387 29092927 PMC5832960

[GR281003HAGC27] Lin CY, Lovén J, Rahl PB, Paranal RM, Burge CB, Bradner JE, Lee TI, Young RA. 2012. Transcriptional amplification in tumor cells with elevated c-Myc. Cell 151: 56–67. 10.1016/j.cell.2012.08.026 23021215 PMC3462372

[GR281003HAGC28] Lindeboom RG, Supek F, Lehner B. 2016. The rules and impact of nonsense-mediated mRNA decay in human cancers. Nat Genet 48: 1112–1118. 10.1038/ng.3664 27618451 PMC5045715

[GR281003HAGC29] Lindeboom RGH, Vermeulen M, Lehner B, Supek F. 2019. The impact of nonsense-mediated mRNA decay on genetic disease, gene editing and cancer immunotherapy. Nat Genet 51: 1645–1651. 10.1038/s41588-019-0517-5 31659324 PMC6858879

[GR281003HAGC30] Litchfield K, Reading JL, Lim EL, Xu H, Liu P, Al-Bakir M, Wong YNS, Rowan A, Funt SA, Merghoub T, 2020. Escape from nonsense-mediated decay associates with anti-tumor immunogenicity. Nat Commun 11: 3800. 10.1038/s41467-020-17526-5 32733040 PMC7393139

[GR281003HAGC31] Liu Z, Dong X, Li Y. 2018. A genome-wide study of allele-specific expression in colorectal cancer. Front Genet 9: 570. 10.3389/fgene.2018.00570 30538721 PMC6277598

[GR281003HAGC32] Lynch TJ, Bell DW, Sordella R, Gurubhagavatula S, Okimoto RA, Brannigan BW, Harris PL, Haserlat SM, Supko JG, Haluska FG, 2004. Activating mutations in the epidermal growth factor receptor underlying responsiveness of non-small-cell lung cancer to gefitinib. N Engl J Med 350: 2129–2139. 10.1056/NEJMoa040938 15118073

[GR281003HAGC33] Mayba O, Gilbert HN, Liu J, Haverty PM, Jhunjhunwala S, Jiang Z, Watanabe C, Zhang Z. 2014. MBASED: allele-specific expression detection in cancer tissues and cell lines. Genome Biol 15: 405. 10.1186/s13059-014-0405-3 25315065 PMC4165366

[GR281003HAGC34] McLaren W, Gil L, Hunt SE, Riat HS, Ritchie GR, Thormann A, Flicek P, Cunningham F. 2016. The Ensembl variant effect predictor. Genome Biol 17: 122. 10.1186/s13059-016-0974-4 27268795 PMC4893825

[GR281003HAGC35] Morin PJ, Sparks AB, Korinek V, Barker N, Clevers H, Vogelstein B, Kinzler KW. 1997. Activation of beta-catenin-Tcf signaling in colon cancer by mutations in beta-catenin or APC. Science 275: 1787–1790. 10.1126/science.275.5307.1787 9065402

[GR281003HAGC36] Palou-Márquez G, Pericot-Masdevall P, Supek F. 2024. Allele-specific expression is selected in tumorigenesis, results from epigenetic changes and has prognostic relevance. bioRxiv 10.1101/2024.09.07.611780

[GR281003HAGC37] PCAWG Transcriptome Core Group. 2020. Genomic basis for RNA alterations in cancer. Nature 578: 129–136. 10.1038/s41586-020-1970-0 32025019 PMC7054216

[GR281003HAGC38] Perera SA, Li D, Shimamura T, Raso MG, Ji H, Chen L, Borgman CL, Zaghlul S, Brandstetter KA, Kubo S, 2009. HER2^YVMA^ drives rapid development of adenosquamous lung tumors in mice that are sensitive to BIBW2992 and rapamycin combination therapy. Proc Natl Acad Sci 106: 474–479. 10.1073/pnas.0808930106 19122144 PMC2626727

[GR281003HAGC39] Pruitt KD, Tatusova T, Maglott DR. 2005. NCBI Reference Sequence (RefSeq): a curated non-redundant sequence database of genomes, transcripts and proteins. Nucleic Acids Res 33: D501–D504. 10.1093/nar/gki025 15608248 PMC539979

[GR281003HAGC40] Robles-Espinoza CD, Mohammadi P, Bonilla X, Gutierrez-Arcelus M. 2021. Allele-specific expression: applications in cancer and technical considerations. Curr Opin Genet Dev 66: 10–19. 10.1016/j.gde.2020.10.007 33383480 PMC7985293

[GR281003HAGC41] Sondka Z, Dhir NB, Carvalho-Silva D, Jupe S, Madhumita, McLaren K, Starkey M, Ward S, Wilding J, Ahmed M, 2024. COSMIC: a curated database of somatic variants and clinical data for cancer. Nucleic Acids Res 52: D1210–D1217. 10.1093/nar/gkad986 38183204 PMC10767972

[GR281003HAGC42] Sherry ST, Ward MH, Kholodov M, Baker J, Phan L, Smigielski EM, Sirotkin K. 2001. dbSNP: the NCBI database of genetic variation. Nucleic Acids Res 29: 308–311. 10.1093/nar/29.1.308 11125122 PMC29783

[GR281003HAGC43] Terekhanova NV, Karpova A, Liang WW, Strzalkowski A, Chen S, Li Y, Southard-Smith AN, Iglesia MD, Wendl MC, Jayasinghe RG, 2023. Epigenetic regulation during cancer transitions across 11 tumour types. Nature 623: 432–441. 10.1038/s41586-023-06682-5 37914932 PMC10632147

[GR281003HAGC44] Turajlic S, Litchfield K, Xu H, Rosenthal R, McGranahan N, Reading JL, Wong YNS, Rowan A, Kanu N, Al Bakir M, 2017. Insertion-and-deletion-derived tumour-specific neoantigens and the immunogenic phenotype: a pan-cancer analysis. Lancet Oncol 18: 1009–1021. 10.1016/S1470-2045(17)30516-8 28694034

[GR281003HAGC45] Van Loo P, Nordgard SH, Lingjærde OC, Russnes HG, Rye IH, Sun W, Weigman VJ, Marynen P, Zetterberg A, Naume B, 2010. Allele-specific copy number analysis of tumors. Proc Natl Acad Sci 107: 16910–16915. 10.1073/pnas.1009843107 20837533 PMC2947907

[GR281003HAGC46] Xu C. 2018. A review of somatic single nucleotide variant calling algorithms for next-generation sequencing data. Comput Struct Biotechnol J 16: 15–24. 10.1016/j.csbj.2018.01.003 29552334 PMC5852328

[GR281003HAGC47] Ye K, Schulz MH, Long Q, Apweiler R, Ning Z. 2009. Pindel: a pattern growth approach to detect break points of large deletions and medium sized insertions from paired-end short reads. Bioinformatics 25: 2865–2871. 10.1093/bioinformatics/btp394 19561018 PMC2781750

[GR281003HAGC48] Zatzman M, Fuligni F, Ripsman R, Suwal T, Comitani F, Edward LM, Denroche R, Jang GH, Notta F, Gallinger S, 2022. Widespread hypertranscription in aggressive human cancers. Sci Adv 8: eabn0238. 10.1126/sciadv.abn0238 36417526 PMC9683723

[GR281003HAGC49] Zhang L, Zhou W, Velculescu VE, Kern SE, Hruban RH, Hamilton SR, Vogelstein B, Kinzler KW. 1997. Gene expression profiles in normal and cancer cells. Science 276: 1268–1272. 10.1126/science.276.5316.1268 9157888

[GR281003HAGC50] Zhang Z, Hernandez K, Savage J, Li S, Miller D, Agrawal S, Ortuno F, Staudt LM, Heath A, Grossman RL. 2021. Uniform genomic data analysis in the NCI Genomic Data Commons. Nat Commun 12: 1226. 10.1038/s41467-021-21254-9 33619257 PMC7900240

[GR281003HAGC51] Zhao Y, Fang LT, Shen TW, Choudhari S, Talsania K, Chen X, Shetty J, Kriga Y, Tran B, Zhu B, 2021. Whole genome and exome sequencing reference datasets from a multi-center and cross-platform benchmark study. Sci Data 8: 296. 10.1038/s41597-021-01077-5 34753956 PMC8578599

[GR281003HAGC52] Zook JM, Chapman B, Wang J, Mittelman D, Hofmann O, Hide W, Salit M. 2014. Integrating human sequence data sets provides a resource of benchmark SNP and indel genotype calls. Nat Biotechnol 32: 246–251. 10.1038/nbt.2835 24531798

