# Supplementary material for: Confounding factors in assessing the enriched expression of somatic mutant alleles in bulk tumor samples
Source: Genome Res. 2026 Apr;36(4):671–83. doi: 10.1101/gr.281003.125 (PMC13138019; doi:10.1101/gr.281003.125)
Supplement: Supplement 1 [file Supplemental_Fig_S1.docx]

**Supplemental Figure S1**

**
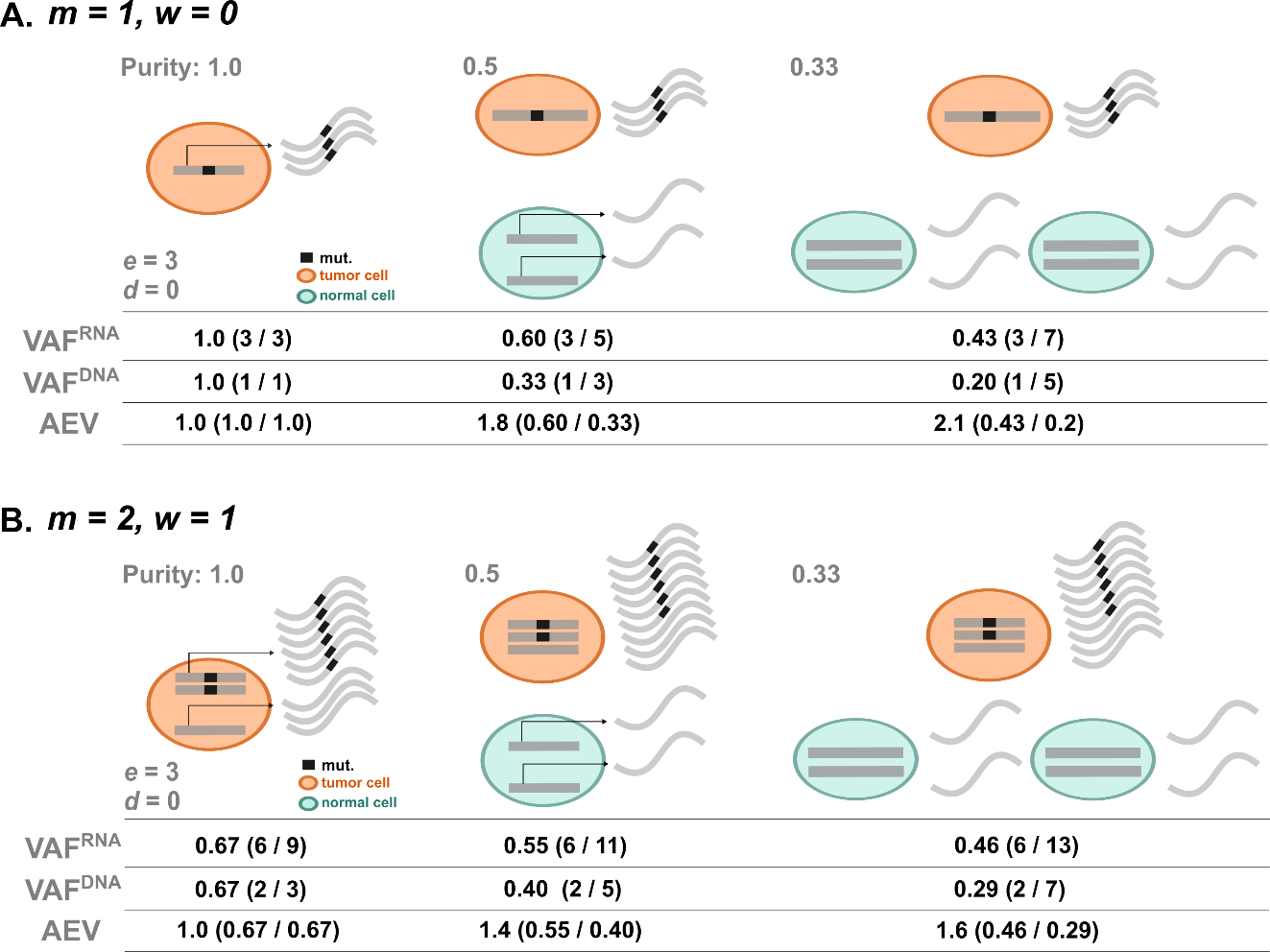
**

**Figure S1. Illustrative examples for non-diploid cases**. **A)** Illustration for a copy loss of wild-type allele case (mutant allele *m* = 1 and wild-type allele *w* = 0). Under the same scenario in main **Fig. 2A** (tumor/normal expression difference *e* = 3 with absence of NMD *d* = 0), the elevation of AEV is shown with the deceasing purity. **B)** Similar illustration for a copy gain of mutant allele case (*m* = 2 and *w* = 1).
