# Supplementary material for: Confounding factors in assessing the enriched expression of somatic mutant alleles in bulk tumor samples
Source: Genome Res. 2026 Apr;36(4):671–83. doi: 10.1101/gr.281003.125 (PMC13138019; doi:10.1101/gr.281003.125)
Supplement: Supplement 2 [file Supplemental_Fig_S2.docx]

**Supplemental Figure S2**

**
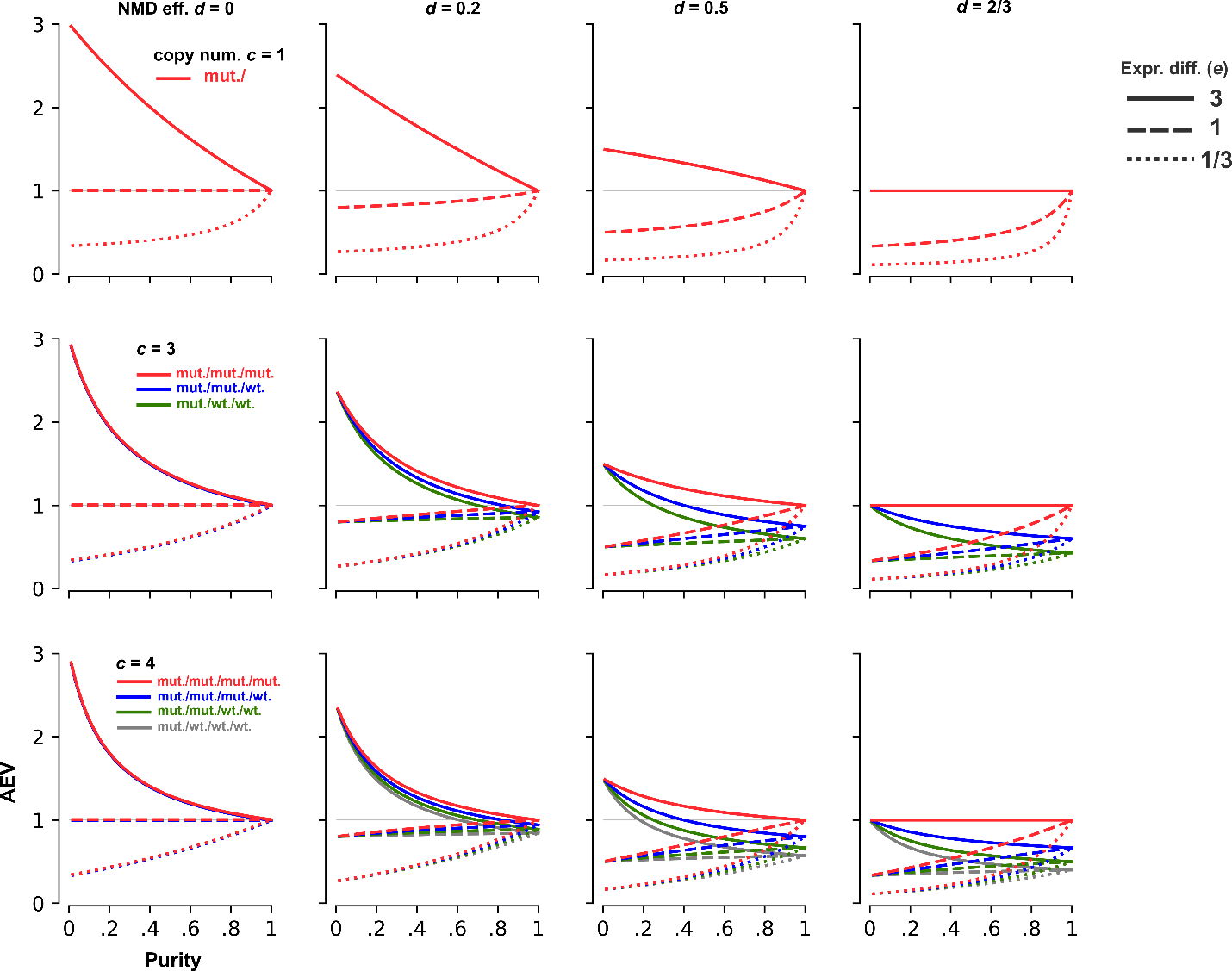
**

**Figure S2. Simulation results of AEV in non-diploid regions.** The AEV model is simulated for copy numbers *c* = 1 (*top*), 3 (*middle*) and 4 (*bottom*) at varying NMD efficiency. Possible mutant allele dosage for each copy number category is labeled by a distinct color. The fourth column illustrates the NMD efficiency *d* = 2/3, representing the upper bound of efficiency to observe an elevated AEV for copy-number adjusted expression difference *e* = 3.
