# Supplementary material for: Confounding factors in assessing the enriched expression of somatic mutant alleles in bulk tumor samples
Source: Genome Res. 2026 Apr;36(4):671–83. doi: 10.1101/gr.281003.125 (PMC13138019; doi:10.1101/gr.281003.125)
Supplement: Supplement 3 [file Supplemental_Fig_S3.docx]

**Supplemental Figure S3**

**
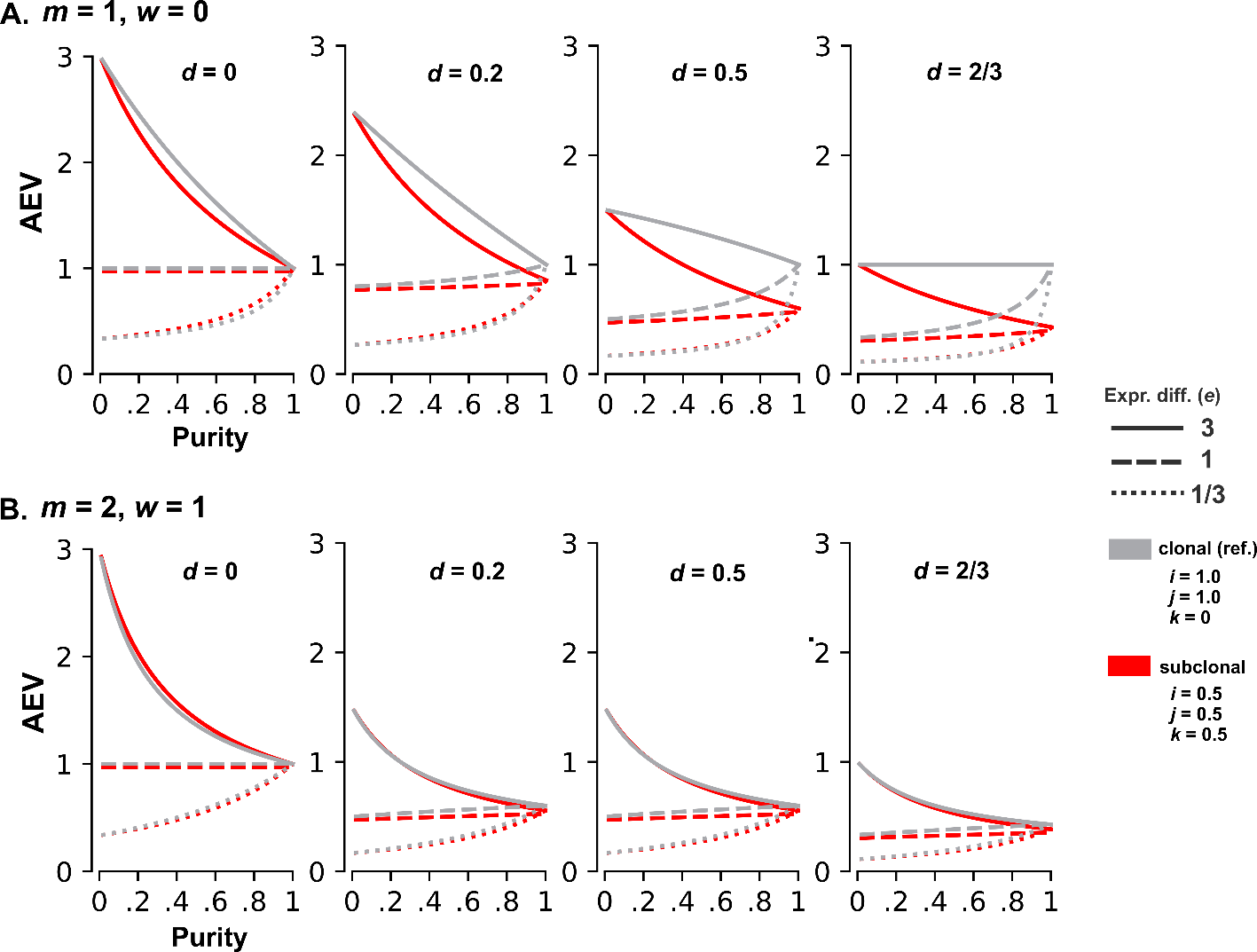
**

**Figure S3. Simulation results of AEV for subclonal CNA/mutation. A)** The AEV model is simulated for a copy loss of wild-type allele (*m* = 1 and *w* = 0) under clonal and subclonal scenarios at varying NMD efficiency and expression difference. The subclonal model has 3 additional parameters to represent subclonality (Equation 8 in METHODS): *i* for fraction of tumor cells carrying CNA, *j* for fraction of tumor cells with CNA carrying the mutant allele, and *k* for fraction of tumor cells without CNA carrying the mutant allele. The subclonal case is plotted in red for *i* = 0.5, *j* = 0.5, *k* = 0.5. The clonal case is added in gray as reference: *i* = 1, *j* = 1, and *k* = 0. Other subclonal scenarios including mutation associated with CNA can be simulated by a custom script (*aev_sim.py*) with similar patterns. **B)** Corresponding simulation for a copy gain of mutant allele case (*m* = 2, w = 1).
