# Supplementary material for: Confounding factors in assessing the enriched expression of somatic mutant alleles in bulk tumor samples
Source: Genome Res. 2026 Apr;36(4):671–83. doi: 10.1101/gr.281003.125 (PMC13138019; doi:10.1101/gr.281003.125)
Supplement: Supplement 4 [file Supplemental_Fig_S4.docx]

**Supplemental Figure S4.**

**
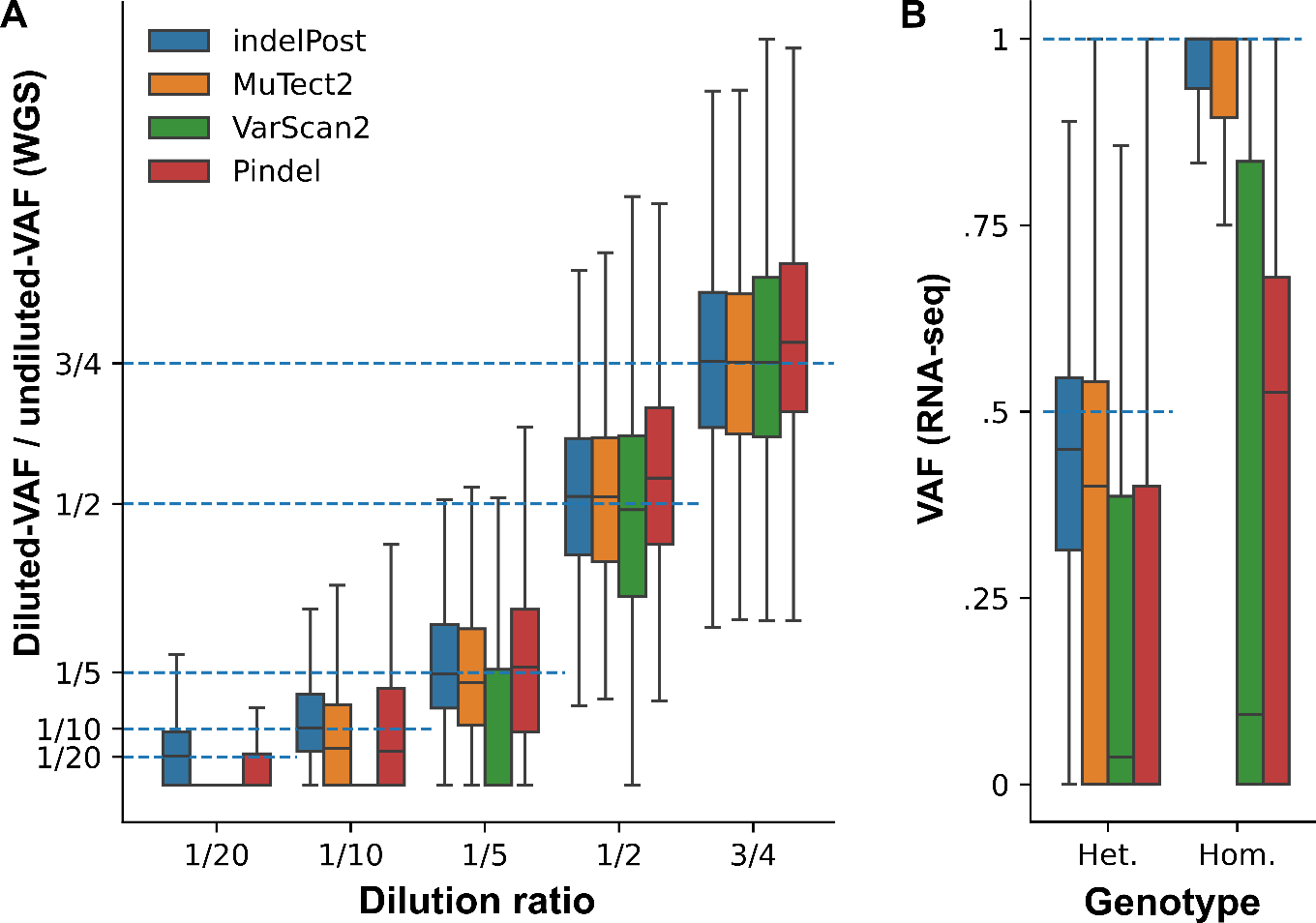
**

**Figure S4.** **Benchmarking read counts for indel allele quantification. A)** Quantification accuracy was compared for indelPost and the indel calling tools used by Genome Data Commons. The reference data sets include somatic indels from a tumor/normal-paired cell line (HCC1395/ HCC1395BL) with whole-genome sequencing (WGS) generated from a serial dilution experiment which has been used for benchmarking of analytical methods. The ground truth value was set as the ratio of VAF measured in the WGS generated from diluted samples divided by VAF in the undiluted WGS sample (see METHODS for detail). Median absolute errors (MAE) are shown in **Supplemental** **Table S1)** Reference germline indel calls from the NA12878 lymphoblastoid cell line were used for benchmark indels in RNA-seq. The ground truth was set to 0.5 for heterozygous (Het.) and 1.0 for homozygous (Hom.) genotypes as established by Genome in a Bottle. To mitigate bias from nonsense-mediated decay (NMD), in-frame indels and indels in untranslated regions (UTRs) were included. Indels in genes with known allele-specific expression (ASE) in this cell line were removed from benchmarking. MAE is shown in **Supplemental Table S2**.
