# Supplementary material for: Confounding factors in assessing the enriched expression of somatic mutant alleles in bulk tumor samples
Source: Genome Res. 2026 Apr;36(4):671–83. doi: 10.1101/gr.281003.125 (PMC13138019; doi:10.1101/gr.281003.125)
Supplement: Supplement 5 [file Supplemental_Fig_S5.docx]

**Supplemental Figure S5.**


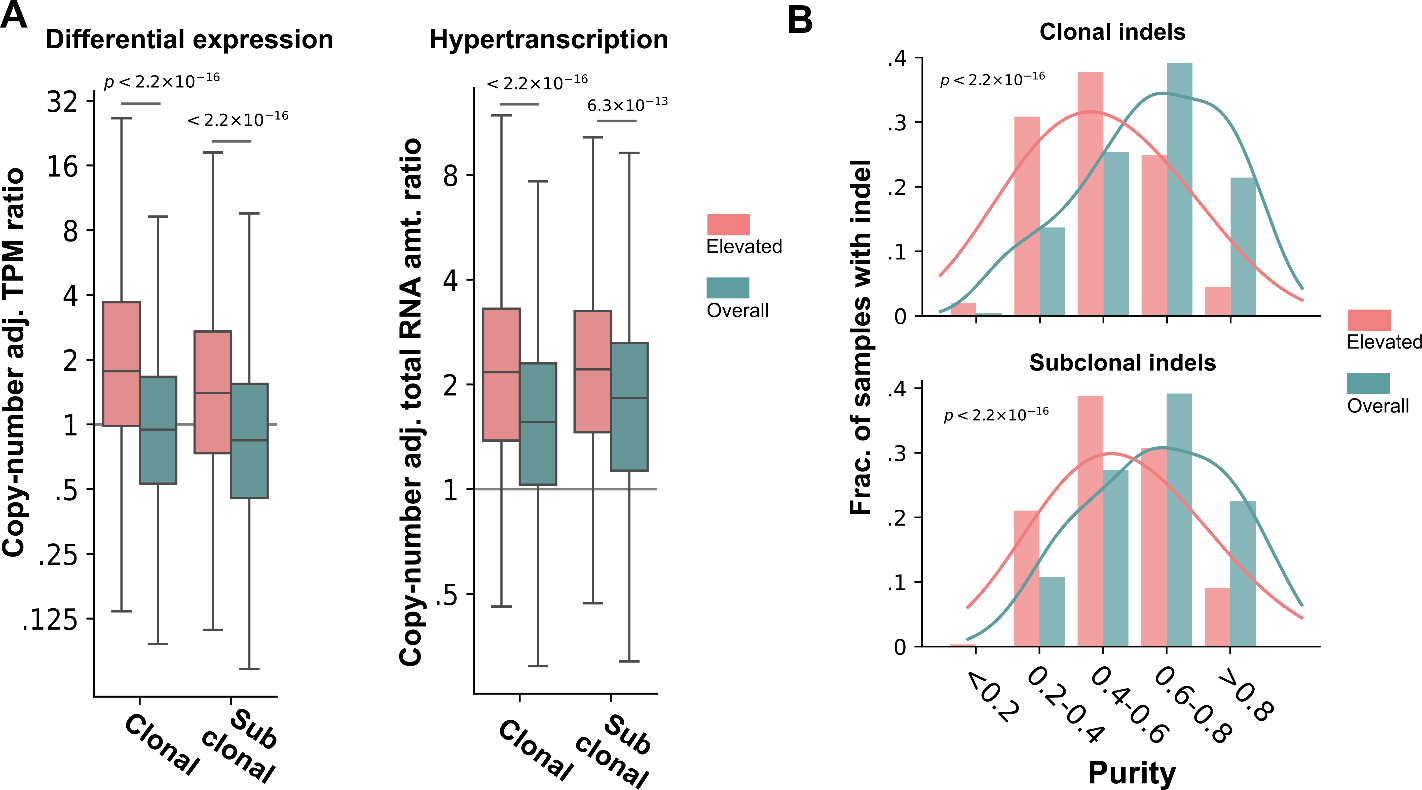


**Figure S5. Evaluation of the AEV model with clonal and subclonal indels. A)** The association between expression differences and AEV (**Fig. 3C** and **D**) was separately reanalyzed for clonal and subclonal indels. **B)** The inverse relationship between purity and AEV (**Fig. 3E**) was separately reanalyzed for clonal (*top*) and subclonal (*bottom*) indels.
