# Supplementary material for: Confounding factors in assessing the enriched expression of somatic mutant alleles in bulk tumor samples
Source: Genome Res. 2026 Apr;36(4):671–83. doi: 10.1101/gr.281003.125 (PMC13138019; doi:10.1101/gr.281003.125)
Supplement: Supplement 6 [file Supplemental_Fig_S6.docx]

**Supplemental Figure S6.**


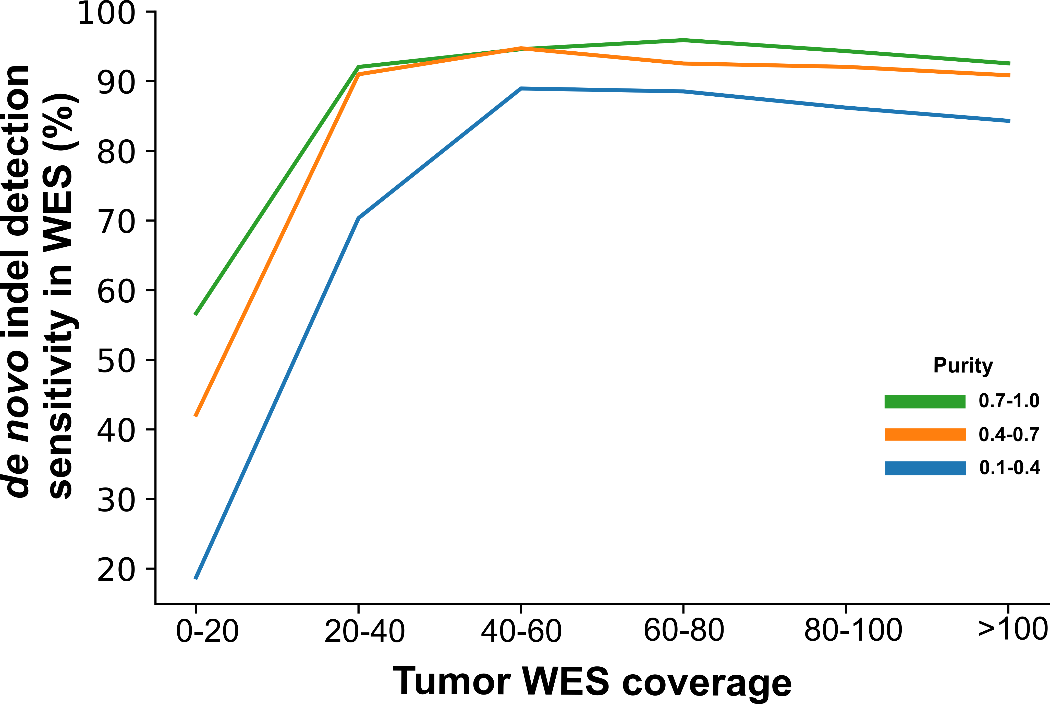


**Figure S6. Assessment of DNA-based indel detection sensitivity stratified by WES coverage and tumor purity.** Somatic driver indels detected by RNA-seq and validated by paired WES (*RNA-only* in **Fig. 5A**) and those detected from paired WES (*DNA-only* and *Shared*) were combined to make a truth set of high-confidence indels. The WES coverage at indel loci and sample’s purity were binned as shown. The sensitivity by the DNA-based approach at each coverage and purity bin was defined by the proportion of *DNA-only* and *Shared* indel at each data point.
