# Supplementary material for: Confounding factors in assessing the enriched expression of somatic mutant alleles in bulk tumor samples
Source: Genome Res. 2026 Apr;36(4):671–83. doi: 10.1101/gr.281003.125 (PMC13138019; doi:10.1101/gr.281003.125)
Supplement: Supplement 7 [file Supplemental_Fig_S7.docx]

**Supplemental Figure S7.**

**
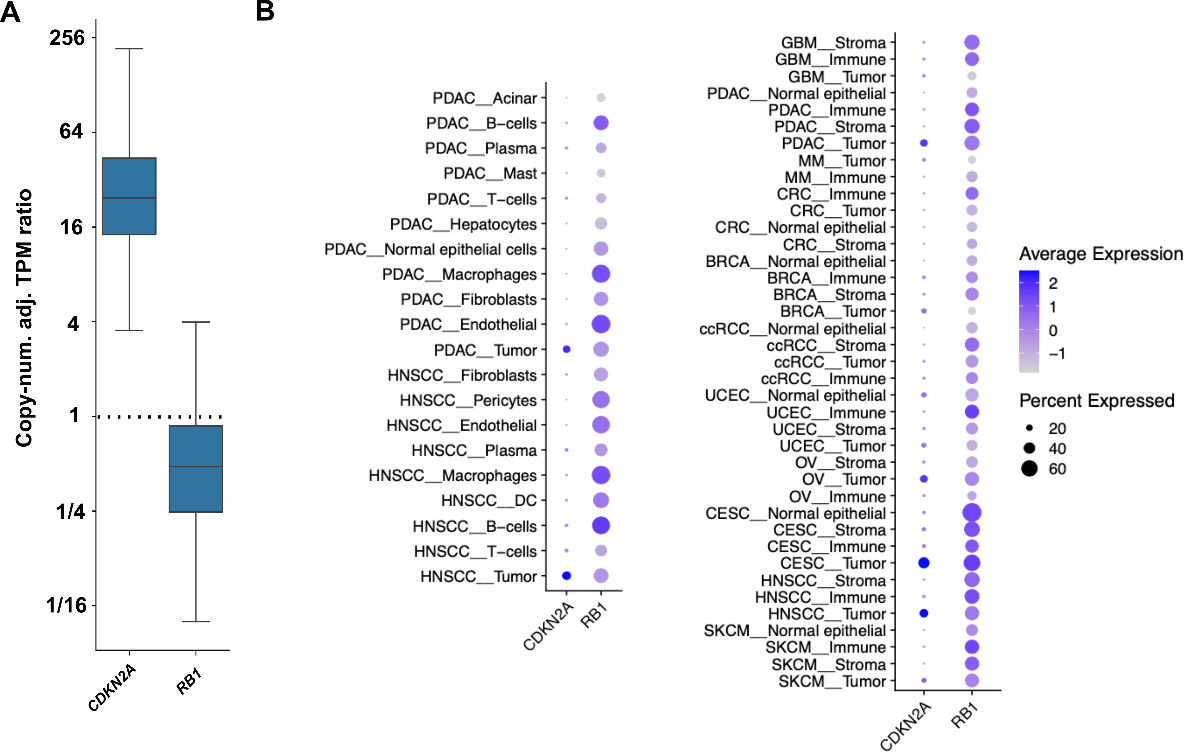
**

**Figure S7. Differential expressions in tumor versus normal cells for *CDKN2A* and *RB1*. A)** The ratio of tumor vs normal expression of the bulk TCGA samples for *CDKN2A* and *RB1* (similar to main **Fig. 3C**). **B)** Expression of *CDKN2A* and *RB1* in tumor and normal cells based on sc/snRNA-seq from a public dataset (METHODS). The size and color of each circle corresponds to the percentage of positive cells and the median expression level, respectively. Left, *CDKN2A* and *RB1* expression in tumor cells and diverse types of normal cells in pancreatic ductal adenocarcinoma (PDAC) and head and neck squamous cell carcinoma (HNSCC), the two cancer types that have the lowest purity. Right, expression in tumor and normal cells across a broad spectrum of cancers. Normal cells are classified into the major categories of stroma, immune and epithelial cells (when applicable).
