# Supplementary material for: Confounding factors in assessing the enriched expression of somatic mutant alleles in bulk tumor samples
Source: Genome Res. 2026 Apr;36(4):671–83. doi: 10.1101/gr.281003.125 (PMC13138019; doi:10.1101/gr.281003.125)
Supplement: Supplement 8 [file Supplemental_Fig_S8.docx]

**Supplemental Figure S8.**

**
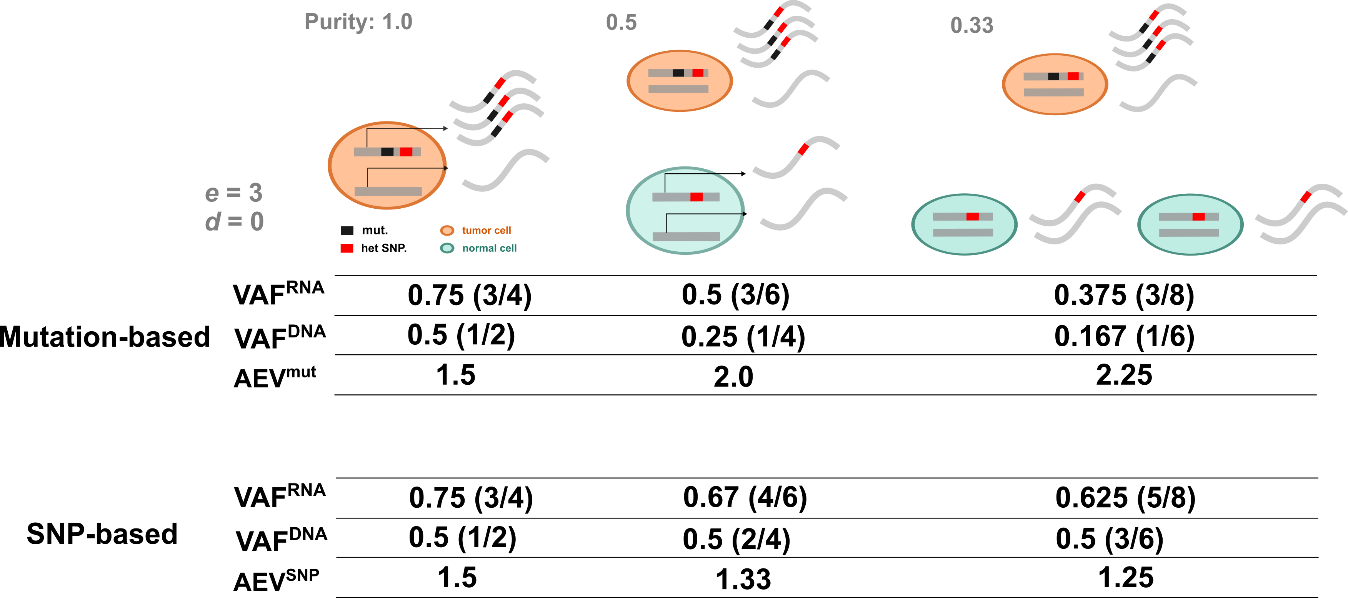
**

**Figure S8. Illustration of mutation-based versus SNP-based allelic imbalance analyses.** In this example, the haplotype harboring the mutant allele is transcribed 3 times more than the wild-type allele in tumor cells (true ASE) and there is no differential expression in the wild-type allele in tumor and normal cells. Allelic imbalance in this scenario is calculated in samples with purity 1.0, 0.5 and 0.33. The mutation-based approach (*top*) uses the read counts of the somatic mutation to calculate the imbalance (AEV) as the ratio of VAF^RNA^ to VAF^DNA^, while the SNP-based approach (*bottom*) uses the heterozygous SNP read counts.
