# Supplementary material for: Confounding factors in assessing the enriched expression of somatic mutant alleles in bulk tumor samples
Source: Genome Res. 2026 Apr;36(4):671–83. doi: 10.1101/gr.281003.125 (PMC13138019; doi:10.1101/gr.281003.125)
Supplement: Supplement 9 [file Supplemental_Fig_S9.docx]

**Supplemental Figure S9**

**
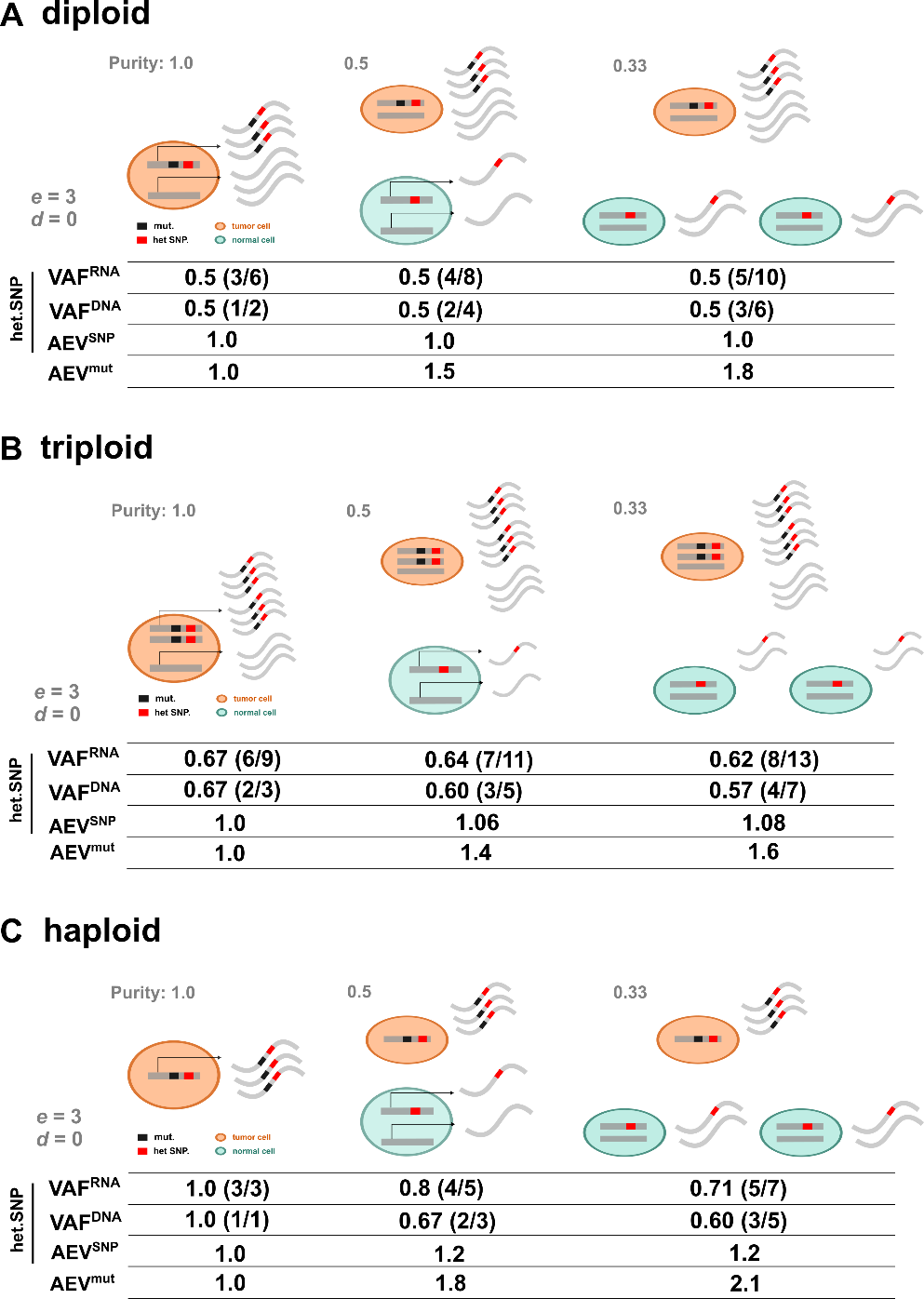
**

**Figure S9. Effect of confounding factors on AEV of heterozygous SNPs.**  Under the scenario that the gene expression per copy is 3 times higher in tumor than in normal (*e* = 3) with absence of NMD (*d* = 0), the SNP-based AEV (AEV^SNP^) is shown at tumor purities 1.0, 0.5, and 0.33 for **A)** diploid, **B)** triploid and **C)** haploid regions. The mutation-based AEV of (AEV^mut^) is shown in the bottom row for comparison.
