# Supplementary material for: Confounding factors in assessing the enriched expression of somatic mutant alleles in bulk tumor samples
Source: Genome Res. 2026 Apr;36(4):671–83. doi: 10.1101/gr.281003.125 (PMC13138019; doi:10.1101/gr.281003.125)
Supplement: Supplement 10 [file Supplemental_Fig_S10.docx]

**Supplemental Figure S10**

**
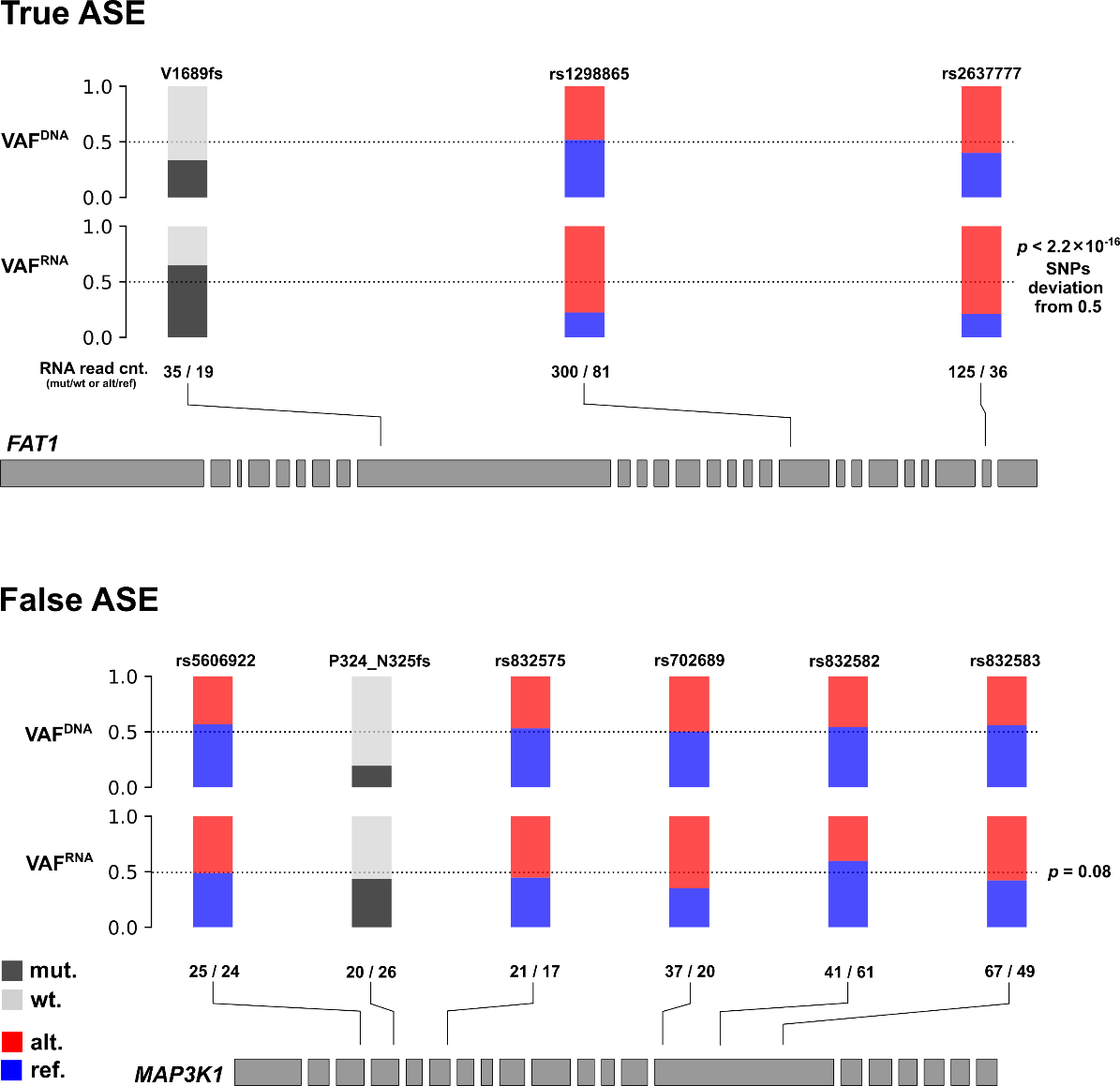
**

**Figure S10. Distinguishing AEV caused by true ASE from that by confounding factors.** The examples used here represent genes in diploid regions. *Top:* true ASE event with *FAT1* V1689fs in a head-and-neck squamous cell carcinoma (TCGA-DQ-5629) characterized by the SNP VAF^RNA^ deviating from 0.5 (excluding mutation VAF^RNA^) with a simulation-based *p* = 2.2×10^-16^ (METHODS). *Bottom*: false ASE with *MAP3K1* P324_N325fs in a breast cancer (TCGA-BH-A0DT) exhibited an insignificant deviation with *p* = 0.08.
