# Supplementary material for: Confounding factors in assessing the enriched expression of somatic mutant alleles in bulk tumor samples
Source: Genome Res. 2026 Apr;36(4):671–83. doi: 10.1101/gr.281003.125 (PMC13138019; doi:10.1101/gr.281003.125)
Supplement: Supplement 11 [file Supplemental_Fig_S11.docx]

**Supplemental Figure S11.**

**
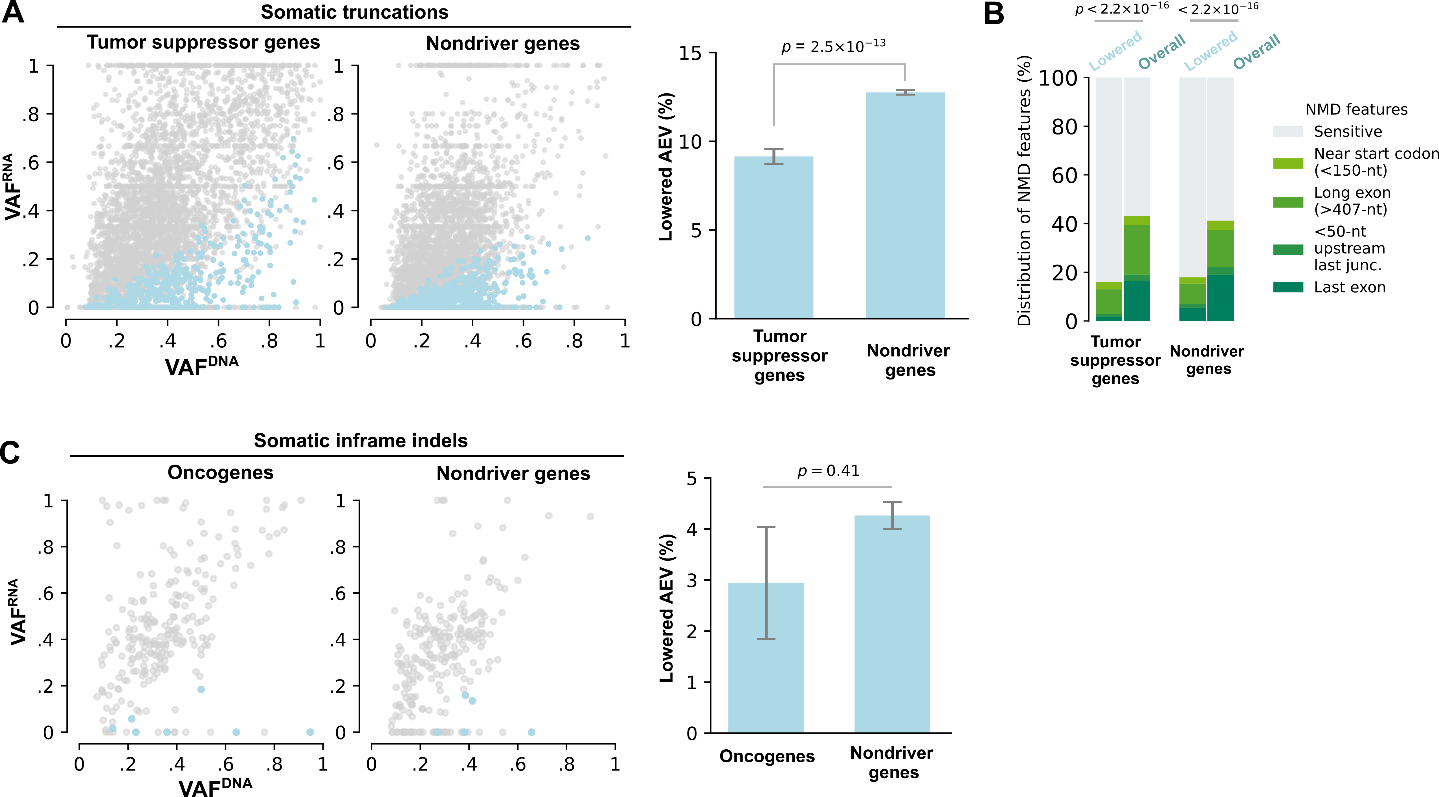
**

**Figure S11. Reduced AEV in TCGA indels. A)** Reduced AEV (VAF ratio < 0.8 with FDR < 0.01) for somatic truncating indels in TSGs (*left*) and non-drivers (*right*). The scatter plot is shown in a format similar to **Fig. 3A** except that reduced AEV cases are shown in blue. **B)** The distribution of NMD features in somatic truncations with reduced AEV was compared to the overall distribution in each gene category (similar to main **Fig. 3G**).**C)** Reduced AEV for somatic in-frame indels in oncogenes and non-drivers in the same format as for elevated AEV shown in **Fig. 4A**.
