# Supplementary material for: Confounding factors in assessing the enriched expression of somatic mutant alleles in bulk tumor samples
Source: Genome Res. 2026 Apr;36(4):671–83. doi: 10.1101/gr.281003.125 (PMC13138019; doi:10.1101/gr.281003.125)
Supplement: Supplement 12 [file Supplemental_Fig_S12.docx]

**Supplemental Figure S12.**

**
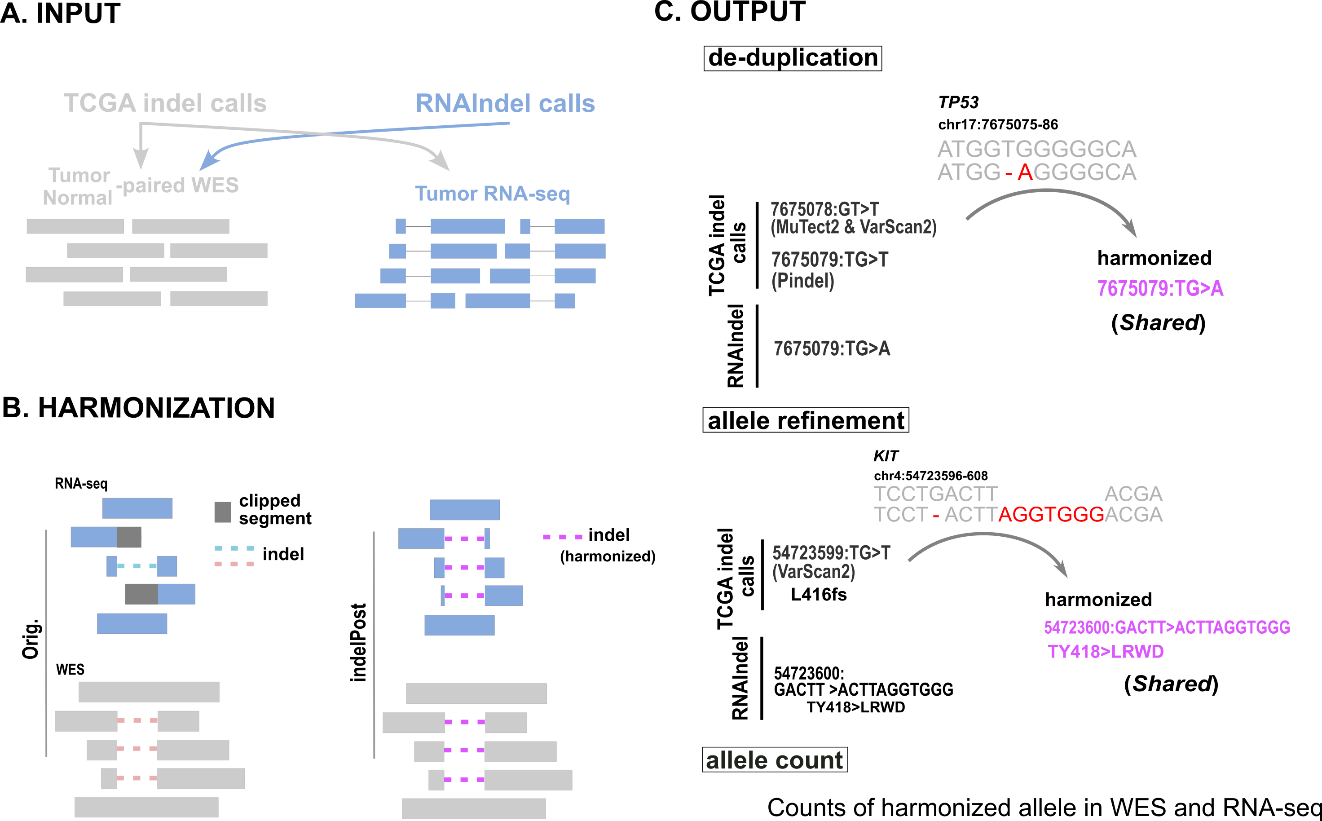
**

**Figure S12. Cross-platform indel comparison.** Somatic indels identified in RNA-seq by RNAIndel and those in WES by the GDC pipeline were harmonized by indelPost for unified representation, which involves the following three steps: **A)** indePost takes the indel calls and BAM files as input. GDC indel calls were processed with the corresponding tumor/normal-paired whole-exome sequencing (WES) and RNA-seq BAM files, while RNA-seq indels were processed with WES because they were internally processed by indelPost in the RNAIndel pipeline. **B)** Indel alignments can be sensitive to the difference in read length (≥75-bp in 99% of TCGA WES vs. 50-bp in 84% of TCGA RNA-seq) and mapping algorithms (BWA for WES vs. STAR for RNA-seq). indelPost harmonizes such inconsistencies via re-alignment, local assembly and read-based phasing to a unified indel representation. **C)** The *TP53* indel was reported in three different representations in a lung squamous cell carcinoma sample (TCGA-NC-A5HT). This was de-duplicated as shown and considered “detected by both DNA and RNA” (*Shared* in **Fig.5A**) because the harmonized allele matched in DNA and RNA. *KIT* is a known oncogene in acute myeloid leukemia (TCGA-AB-2846) and in-frame indels at this amino acid position are expected. The TCGA frameshift allele by VarScan2 (not reported by other detectors) was corrected to an expected in-frame allele by indelPost. While RNAIndel’s report was correct, this in-frame indel was similarly classified as “*Shared*”. Allele counts based on harmonized allele in WES and RNA-seq were used throughout this current study (see benchmark result in **Supplemental** **Fig. S4**). A custom script using indelPost for this workflow is provided as (*cross_platform_checker.py*).
