# Supplementary material for: Confounding factors in assessing the enriched expression of somatic mutant alleles in bulk tumor samples
Source: Genome Res. 2026 Apr;36(4):671–83. doi: 10.1101/gr.281003.125 (PMC13138019; doi:10.1101/gr.281003.125)
Supplement: Supplement 13 [file Supplemental_Table_S1.docx]

**Supplemental Table S1.**

| Dilution | indelPost | | MuTect2 | | VarScan2 | | Pindel | |
| --- | --- | --- | --- | --- | --- | --- | --- | --- |
| 3/4 | 0.754 | (0.118) | 0.753 | (0.124) | 0.752 | (0.140) | 0.787 | (0.136) |
| 1/2 | 0.513 | (0.106) | 0.513 | (0.108) | 0.490 | (0.140) | 0.546 | (0.128) |
| 1/5 | 0.197 | (0.071) | 0.182 | (0.826) | 0.0 | (0.200) | 0.210 | (0.128) |
| 1/10 | 0.102 | (0.048) | 0.065 | (0.100) | 0.0 | (0.100) | 0.060 | (0.100) |
| 1/20 | 0.051 | (0.044) | 0.0 | (0.050) | 0.0 | (0.050) | 0.0 | (0.050) |

**Table S1. Allele quantification benchmarking (DNA).** Median VAF ratio and median absolute error (in parentheses) are shown at each dilution.
