# Supplementary material for: Confounding factors in assessing the enriched expression of somatic mutant alleles in bulk tumor samples
Source: Genome Res. 2026 Apr;36(4):671–83. doi: 10.1101/gr.281003.125 (PMC13138019; doi:10.1101/gr.281003.125)
Supplement: Supplement 14 [file Supplemental_Table_S2.docx]

**Supplemental Table S2.**

| Genotype | indelPost | | MuTect2 | | VarScan2 | | Pindel | |
| --- | --- | --- | --- | --- | --- | --- | --- | --- |
| Het. | 0.450 | (0.125) | 0.400 | (0.183) | 0.037 | (0.474) | 0.000 | (0.5) |
| Hom. | 1.0 | (0.0) | 1.0 | (0.0) | 0.094 | (0.906) | 0.526 | (0.474) |

**Table S2. Allele quantification benchmarking (RNA).** Median VAF ratio and median absolute error (in parentheses) are shown for the ground truth genotypes. Het.: heterozygous (true VAF = 0.5). Hom.: homozygous (true VAF = 1.0)
