# Supplementary material for: Confounding factors in assessing the enriched expression of somatic mutant alleles in bulk tumor samples
Source: Genome Res. 2026 Apr;36(4):671–83. doi: 10.1101/gr.281003.125 (PMC13138019; doi:10.1101/gr.281003.125)
Supplement: Supplement 18 [file Supplemental_Table_S6.docx]

**Supplemental Table S6.**

|  |  | version 2.2.3 | | version 3.0.1 | |
| --- | --- | --- | --- | --- | --- |
| Case ID | BAM file size | Run time | Cost | Run time | Cost |
| TCGA-CZ-5465 | 12.3 GB | 356 min | 1.74 USD | 29 | 0.25 |
| TCGA-A4A57E | 5.2 | 86 | 0.53 | 11 | 0.09 |
| TCGA-CZ-5454 | 11.5 | 439 | 2.11 | 36 | 0.31 |
| TCGA-B9-4115 | 8.6 | 212 | 1.04 | 20 | 0.17 |
| TCGA-BQ-7059 | 10.3 | 365 | 1.79 | 40 | 0.34 |
| TCGA-BQ-7055 | 6.2 | 79 | 0.39 | 9 | 0.08 |
| TCGA-BQ-7045 | 3.7 | 42 | 0.26 | 7 | 0.06 |
| TCGA-CZ-5455 | 13.1 | 253 | 1.57 | 36 | 0.25 |
| TCGA-B0-5699 | 7.7 | 205 | 0.9 | 22 | 0.14 |
| TCGA-B0-4700 | 8.8 | 134 | 0.83 | 15 | 0.11 |

**Table S6. Cost performance improvement by RNAIndel version 3.** From renal clear cell carcinoma (TCGA KIRC), 10 RNA-seq samples were selected ranging from 3.7 - 13.1 gigabyte (GB) data size. Refactored RNAIndel (version 3.0.1) [10] was compared to the original version (2.2.3) on Cancer Genomics Cloud [11]. The compute cost is based on the pricing as of Apr. 2021. USD: United States dollar.
